# Transcriptome Analysis in Venom Gland of the Predatory Giant Ant *Dinoponera quadriceps*: Insights into the Polypeptide Toxin Arsenal of Hymenopterans

**DOI:** 10.1371/journal.pone.0087556

**Published:** 2014-01-31

**Authors:** Alba F. C. Torres, Chen Huang, Cheong-Meng Chong, Siu Wai Leung, Álvaro R. B. Prieto-da-Silva, Alexandre Havt, Yves P. Quinet, Alice M. C. Martins, Simon M. Y. Lee, Gandhi Rádis-Baptista

**Affiliations:** 1 Department of Clinical and Toxicological Analysis, Faculty of Pharmacy, Federal University of Ceara, Fortaleza, Ceara, Brazil; 2 State Key Laboratory of Quality Research in Chinese Medicine and Institute of Chinese Medical Sciences, University of Macau, Macao, China; 3 Laboratory of Genetics, Institute Butantan, Sao Paulo, Brazil; 4 Department of Physiology and Pharmacology, Faculty of Medicine, Federal University of Ceara, Fortaleza, Ceara, Brazil; 5 Laboratory of Entomology, State University of Ceara, Fortaleza, Ceara, Brazil; 6 Laboratory of Biochemistry and Biotechnology, Institute for Marine Sciences, Federal University of Ceara, Brazil; Instituto Butantan, Brazil

## Abstract

**Background:**

*Dinoponera quadriceps* is a predatory giant ant that inhabits the Neotropical region and subdues its prey (insects) with stings that deliver a toxic cocktail of molecules. Human accidents occasionally occur and cause local pain and systemic symptoms. A comprehensive study of the *D. quadriceps* venom gland transcriptome is required to advance our knowledge about the toxin repertoire of the giant ant venom and to understand the physiopathological basis of Hymenoptera envenomation.

**Results:**

We conducted a transcriptome analysis of a cDNA library from the *D. quadriceps* venom gland with Sanger sequencing in combination with whole-transcriptome shotgun deep sequencing. From the cDNA library, a total of 420 independent clones were analyzed. Although the proportion of dinoponeratoxin isoform precursors was high, the first giant ant venom inhibitor cysteine-knot (ICK) toxin was found. The deep next generation sequencing yielded a total of 2,514,767 raw reads that were assembled into 18,546 contigs. A BLAST search of the assembled contigs against non-redundant and Swiss-Prot databases showed that 6,463 contigs corresponded to BLASTx hits and indicated an interesting diversity of transcripts related to venom gene expression. The majority of these venom-related sequences code for a major polypeptide core, which comprises venom allergens, lethal-like proteins and esterases, and a minor peptide framework composed of inter-specific structurally conserved cysteine-rich toxins. Both the cDNA library and deep sequencing yielded large proportions of contigs that showed no similarities with known sequences.

**Conclusions:**

To our knowledge, this is the first report of the venom gland transcriptome of the New World giant ant *D. quadriceps*. The glandular venom system was dissected, and the toxin arsenal was revealed; this process brought to light novel sequences that included an ICK-folded toxins, allergen proteins, esterases (phospholipases and carboxylesterases), and lethal-like toxins. These findings contribute to the understanding of the ecology, behavior and venomics of hymenopterans.

## Introduction

In recent years, the order Hymenoptera, which comprises numerous species of bees, wasps and ants, has received increasing attention because of their direct and indirect influences on human health, ecological balance, agriculture and the forestry economy. The importance of investigating hymenopterans is well-known; thus, information from diverse studies has culminated with the establishment of a genome database (http://hymenopteragenome.org). Presently, this database maintains scientific information about one species of bee (*Apis mellifera*), two species of bumblebees (*Bombus terrestris* and *B. impatiens*), a parasitoid wasp (*Nasonia vitripennis*), and seven species of ants [1]. The seven ant species detailed in this database include the fungus-growing (leaf cutter) ants *Acromyrmex echinatior* and *Atta cephalotes*, the Florida carpenter ant *Camponotus floridanus*, Jerdon's jumping ant *Harpegnathos saltator*, the Argentine ant *Linepithema humile*, the red harvester ant *Pogonomyrmex barbatus*, and the fire ant *Solenopsis invicta* (http://hymenopteragenome.org/ant_genomes/).

From the perspective of molecular toxinology and venomics, scientific interest and public concerns about animal venoms have sharply increased due the vast diversity of bioactive compounds, the safety hazards, and the potential therapeutic uses of isolated compounds [2]. Hymenopterans offer a myriad of biologically active molecules that incorporate the physiopathological bases of the causes of ant venom allergies and envenomation and the traditional and modern uses of ants for medicines and in programs of drug discovery. For example, in countries of East Asia, Africa and South America, ant venoms have been used to treat rheumatoid arthritis [3]. Badr and collaborators [4] demonstrated that the crude venom from *Pachycondyla senaarensis* induces death by apoptosis in breast carcinoma cells. The same venom has been proven to display anti-inflammatory and anti-oxidant properties [5]. Additionally, the venom of Chinese medicinal ant *Polyrhachis lammellidens* elicits analgesic and anti-inflammatory effects [6]. Peptides from *Pachycondyla goeldii* and *Myrmecia* sp. have been demonstrated to be effective against Gram-negative and Gram-positive bacteria, including the methicilin-resistant *Staphyloccocus aureus* and the pathogenic fungus *Candida albicans* [7], [8]. Moreover, the antimicrobial effects of crude venom from *Crematogaster pygmaea*, which is composed of electrophilic aldehydes, have recently been demonstrated [9].

Our group has been investigating the biological effects of *D. quadriceps* crude venom in different experimental models. In a system of isolated perfused rat kidney, *D. quadriceps* venom triggers increases in urinary flow, decreases in vascular resistance, and reductions in sodium tubular transport; these findings demonstrate the natriuretic and diuretic-induction effects of this crude venom [10]. In pentylenetetrazol-induced seizure models, this giant ant venom exhibits neuroprotective activities that depend on the route of administration [11], [12]. Additionally, models of antinociception, including those evoked by formalin, writhing, von Frey fibers and hot plates, the analgesic effects of *D. quadriceps* crude venom have been verified to be efficacious [13].

**Figure 1:**
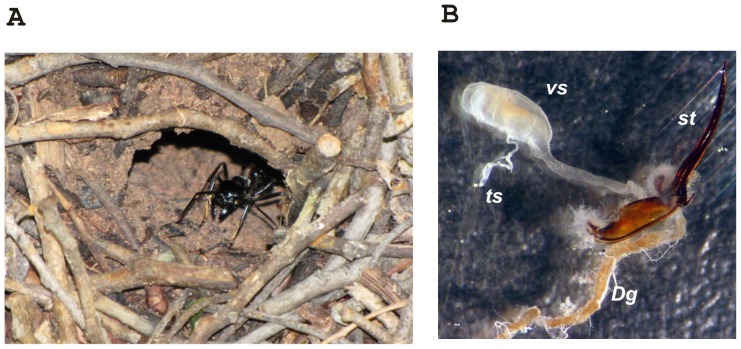
Dinoponera quadriceps in the field and its dissected venom apparatus. Part A - A single specimen of D. quadriceps protecting the nest's entrance. Part B - Dissected *D. quadriceps* venom apparatus (×40). Abbreviations: *Dg* - Doffur's gland; *cv* - convoluted gland (not observable); *st* - secretory tubule; *vs*- venom sac; sg – sting.

The genus *Dinoponera* (Hymenoptera: Vespoidea: Formicidae) belongs to the subfamily Ponerinae and comprises the following eight species with strict Neotropical distributions [14]–[16]: *D. australis*, *D. gigantea*, *D. hispida*, *D. longipes*, *D. lucida*, *D. mutica*, *D. quadriceps*, and *D. snellingi*. Collectively, these species form a group of primitive poneromorphs ants with body sizes ranging from 3 to 4 cm, which places them among the largest ants in the world [17], [18]. Along with some other ponerine genera, *Dinoponera* ants have queenless colonies, and reproductive functions are performed by one mated dominant worker called a gamergate [19]. In Brazil, *Dinoponera* ants, popularly called “falsas tocandiras”, exemplify the genera with strict New World distributions. The giant ant *D. quadriceps* preferentially inhabits the Brazilian northeast and can be found in a variety of vegetation types including *caatinga* (a xerophyllous vegetation), *cerrado* (a savannah-like vegetation), *brejos de altitude* (humid mountain forests) and Atlantic forest. *D. quadriceps* nests (Fig. 1-A) have an average population of 60 workers, one or two entrances, a vertical gallery that is between 30 to 120 cm deep, and up to 16 chambers [15], [20]. As in all Aculeata hymenopterans for which anatomical observations exist, the venom gland of *Dinoponera* is formed by free tubules, a convoluted gland and a reservoir (venom sac) (Fig. 1-B) [21], [22]. The free tubules and convoluted gland are involved in venom production, and the venom is a fluid that is rich in polypeptides and characteristic of the stinging ants that belong to the subfamilies Myrmicinae, Ponerinae and Pseudomyrmecinae [23].

*Dinoponera* ants preferentially prey on insects and small arthropods, but inevitable interactions with humans occur, and, upon venom injection, the location of the sting becomes extremely painful. Additionally, the stings may have systemic manifestations such as sweating, nausea, vomiting, fever, lymphadenopathy, cardiac arrhythmias and tissue damage [24]–[27]. These symptoms are mostly attributable to a cocktail of low molecular weight organic compounds and pharmacologically active polypeptides that is present in the venom. Indeed, regarding the polypeptide core of the venom, short peptides isolated from *Paraponera clavata* venom (i.e., poneratoxins (PoTx)) have been proven to prolong the action of sodium channels and interfere with neurotransmission [28], [29]. To identify individual venom peptides and proteins in the venom of *Dinoponera australis*, Johnson and colleagues [30] conducted a proteomic survey via high performance liquid chromatography coupled with mass-spectrometry (HPLC/DAD/MS). However, only a limited number of peptides and related partial protein fragments were fully characterized. Consequently, a comprehensive description of the toxic polypeptide repertoire of the venom of the *Dinoponera* giant ant does not yet exist at either the genus or species level. Moreover, despite the large amounts of information about the compositions of the venoms of some species of hymenopterans of the family Formicidae that are available, information about the effects of the components of the venoms on biological systems and the potential uses of the individual polypeptides to manipulate cell biology are still scarce and deserving of deeper investigation. Given this context and the ecological and pharmacological interest in *D. quadriceps* venomics, in this study we report on the transcriptome of the giant ant venom gland and present new data that should further our comprehension of the evolution of the toxin arsenal of Hymenoptera.

## Results

### Sanger sequencing and cluster assemblies

**Figure 2:**
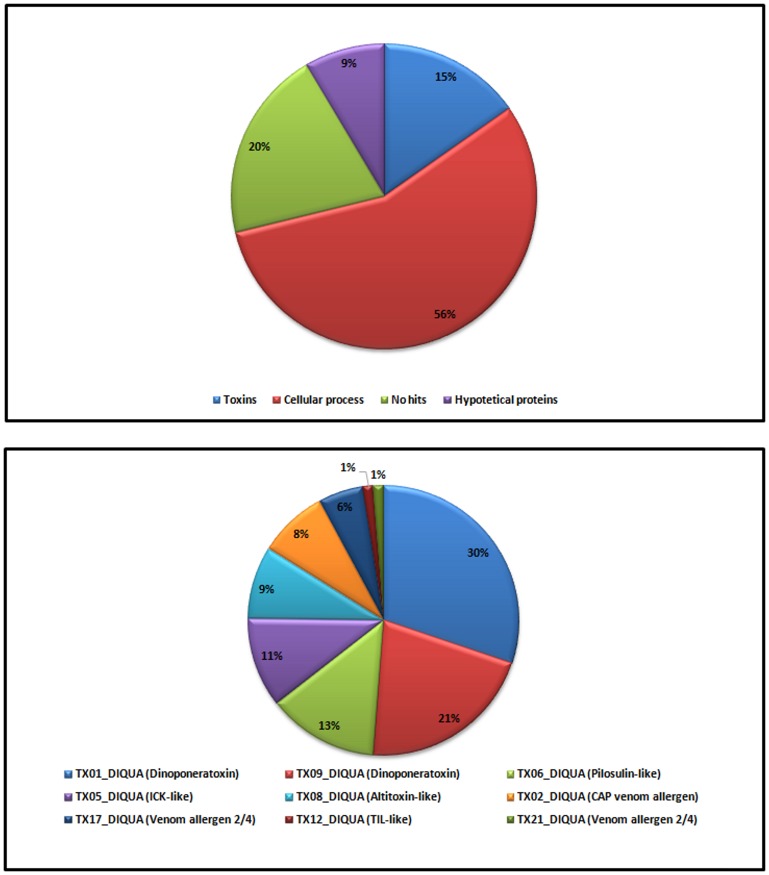
Overall classification of ESTs from the *D. quadriceps* venom gland cDNA (ESTs) library based on function. EST sequences were annotated by comparing contigs and singlets (E-values≤1.0E-5) with the non-redundant Genbank translated protein database using BLASTX. Search stringency was eventually adjusted (E-values lower than 1.0E-5) and database restricted to hymenopterans with the aim of finding relevant familial- and structural-related sequences of hymenopterans toxins. Part A – relative number of polypeptides which were counted as individual random sequenced clones. The group of <cellular process> refers to enzymes and polypeptides involved in the venom gland metabolism. Part B – relative numbers of venom-related sequences found in the overrepresented small toxin dataset. Under the term <toxin> is included dinoponeratoxins, venom allergens, and venom cysteine rich peptides (part B).

A full-length cDNA library was constructed with approximately 20 venom glands from female (wingless) *D. quadriceps*. A cloning efficiency of 1×10^5^ cfu/µg DNA was achieved, and the average insert size was 563 bp. A total of 420 individual randomly selected clones were sequenced by with the dideoxy chain-termination (Sanger) method. Nucleotide sequences with greater base calling values were processed via biocomputation (CLC Main Workbench v. 6.8.4, CLC Bio, U.S.A.). The high-quality trimmed ESTs generated an assemblage of 22 contigs and 44 singletons. As summarized in figure 2, the comparison of translated sequences with non-redundant protein databanks using BlastX analysis allowed for the classification and identification the following clusters (Fig. 2-A): (1) proteins involved in general metabolism (56% of contigs and singlets; e.g., transferases, ATP synthase, dehydrogenases, ribosomal proteins, and cytocrome c); (2) venom-linked polypeptide gene sequences (15%) that highlighted the predominant expression of dinoponeratoxins, allergen peptides, and an ICK motif-containing toxin; (3) cDNA precursor sequences that yielded no hits (20%), and (4) ESTs that represented hypothetical proteins with unknown function (5%). Comparisons of the amino acid and nucleotide sequences of the hypothetical proteins with a specific arthropod database revealed that the ESTs of *D. quadriceps* shared homology with polypeptides from scorpions and others ants, including *H. saltator*, *S. invicta* and *S. saevissima* (Fig. 2-B; Table S1).

### Ion Torrent deep RNA sequencing and read assembly

**Figure 3:**
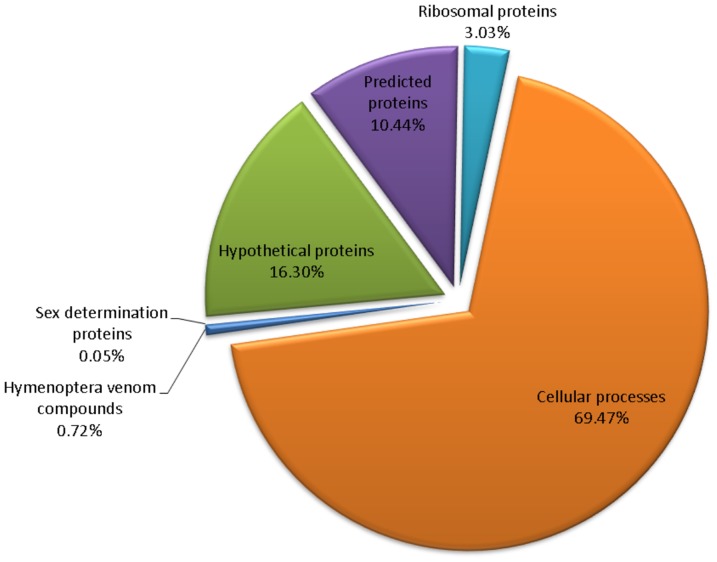
General overview of the transcripts in the *D. quadriceps* venom gland identified by deep RNA sequencing. Annotation of contigs from the RNA-Seq assembly of the giant ant venom following the conventions of figure 2; i.e., the E-value cut-off was at least 1.0E-5 for BLASTX functional comparison.

**Figure 4:**
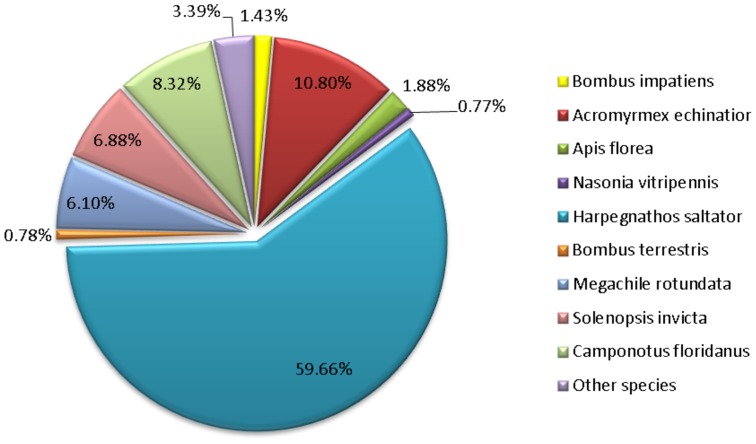
Distribution of the Hymenoptera species as determined by best protein hits. The percentages of homologs from distinct hymenopterans species with which the 6,429 contigs of the *D. quadriceps* venom gland shared the high sequence similarities.

Next generation RNA-sequencing of the same *D. quadriceps* venom gland starting material that was used to construct the cDNA library generated a total of 2,514,767 raw reads (average read length = 230 bps). The sequencing data were subjected in-depth bioinformatics analyses. Initially, the raw reads were filtered by an in-house Perl script to remove low quality reads, which resulted in a total of 18,546 assembled transcripts with contig sizes ranging from 101 to 8114 bp, and an average transcript length of 232.09 bp (Table S2). The size distribution of the assembled transcripts is shown in Figure S1. The processed contigs were annotated by querying the non-redundant (nr) protein database via transcript sequence comparison using the BLASTx algorithm, and 6429 contigs with high gene homology were retrieved (Table 1 and Fig. 3). The majority of *D. quadriceps* transcript sequences (3807 contigs) were matched to those of another ant species (*H. saltator*), which reflects their close phylogenetic relationship of these species. Interestingly, some contigs showed high sequence similarity with Hymenoptera species such as *A. florae* and *Megachile rotundata* (see Fig. 4 for details). Nearly all (∼98%) EST sequences retrieved from cDNA library matched and strongly supported the assembled contigs that were obtained by next generation RNA-sequencing. Based on non-redundant annotation information, these RNA-derived contigs were then categorized into protein families involved in, for example, cellular processes, Hymenoptera venom-related polypeptides and ribosomal proteins (Fig. 3). This Transcriptome Shotgun Assembly project has been deposited at DDBJ/EMBL/GenBank under the accession GANS00000000. The version described in this paper is the first version, GANS01000000.

**Table 1:** Summary of assembled contigs derived from RNA sequencing and comparison with the ESTs of the *D. quadriceps* venom gland transcriptome.

Total number of contigs	18546
(Average length, bp)	(232)
BLASTx-hits	6429
BLASTx-hits supported ESTs	32
No-BLASTx-hits	12117
No-BLASTx-hits supported ESTs	16

### Annotation of transcript clusters related to *D. quadriceps* venomics

**Figure 5:**
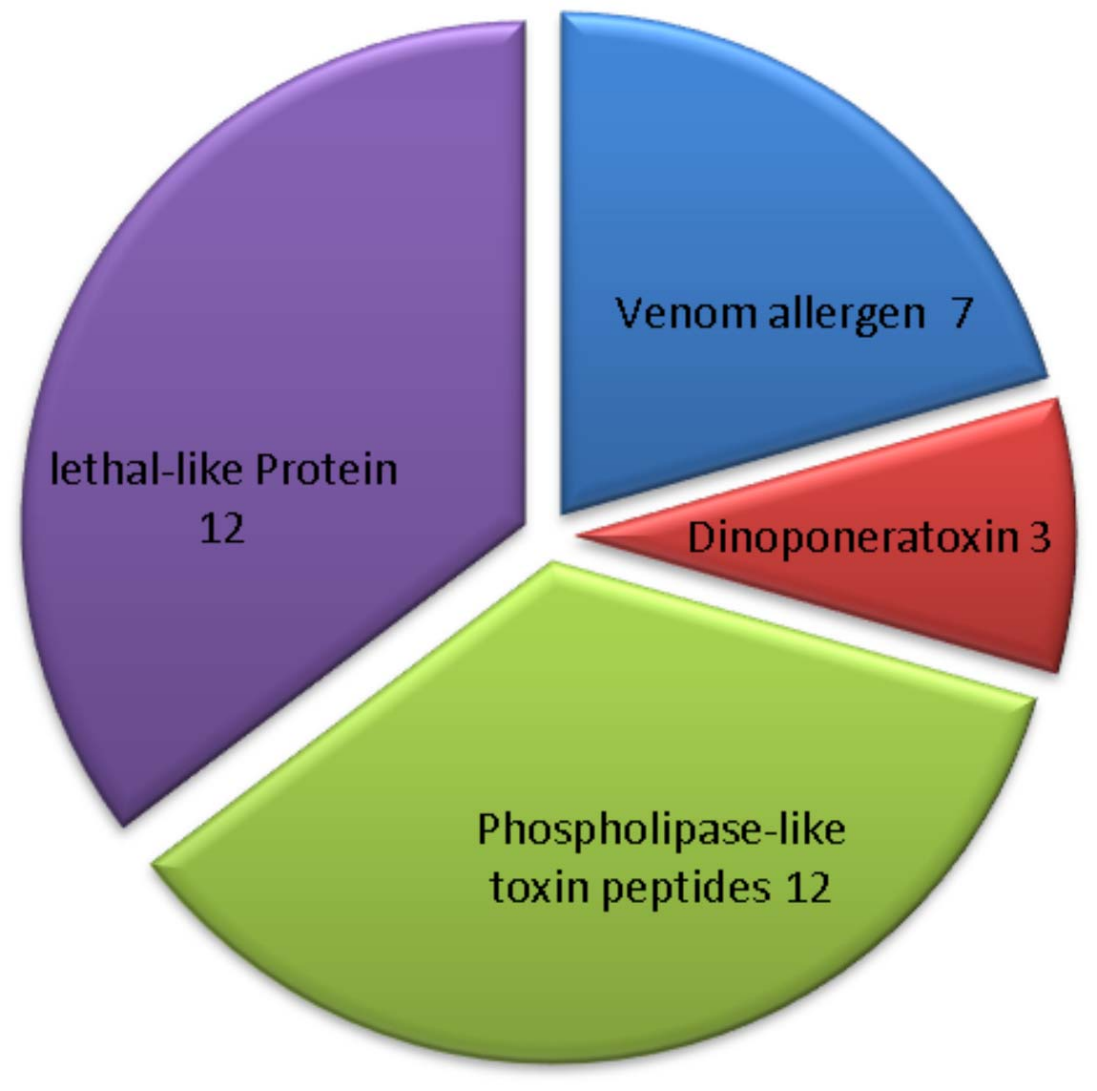
Proportions of transcript toxin and toxin-related components expressed in the venom gland of *D. quadriceps*. Toxins and venom-related protein precursors represented less than 1% of all transcripts (18,524) in the giant ant venome. As shown, the major toxin core was composed of four groups of polypeptides.

**Figure 6:**
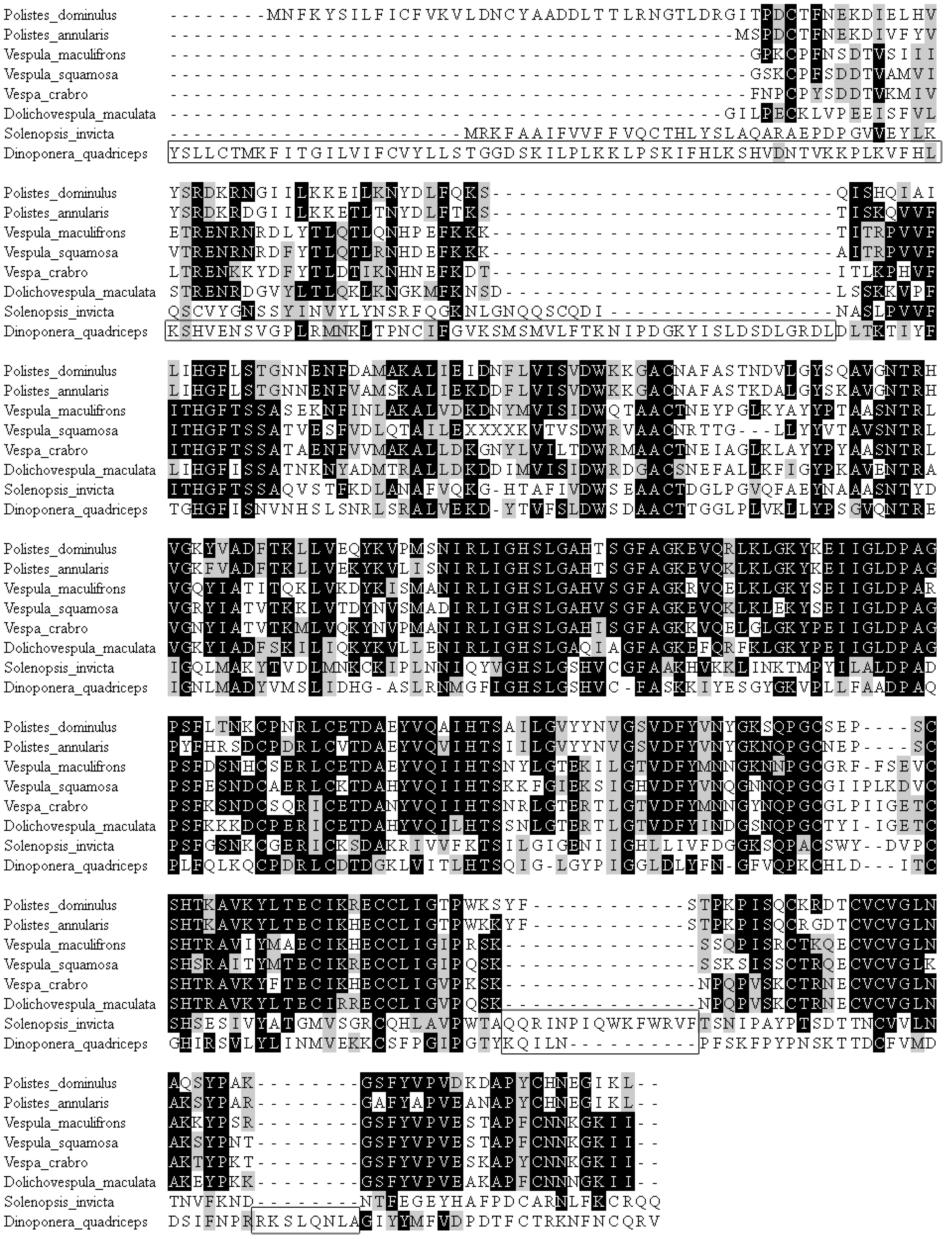
Multiple alignments of the deduced amino acid sequences of venom allergen I (Sol i 1) from *D. quadriceps* with known sequences from different ant and wasp species. The identical and conserved amino acid residues of diverse ant and wasp hymenopterans are highlighted in black and gray, respectively. Dots represent gaps.

**Figure 7:**
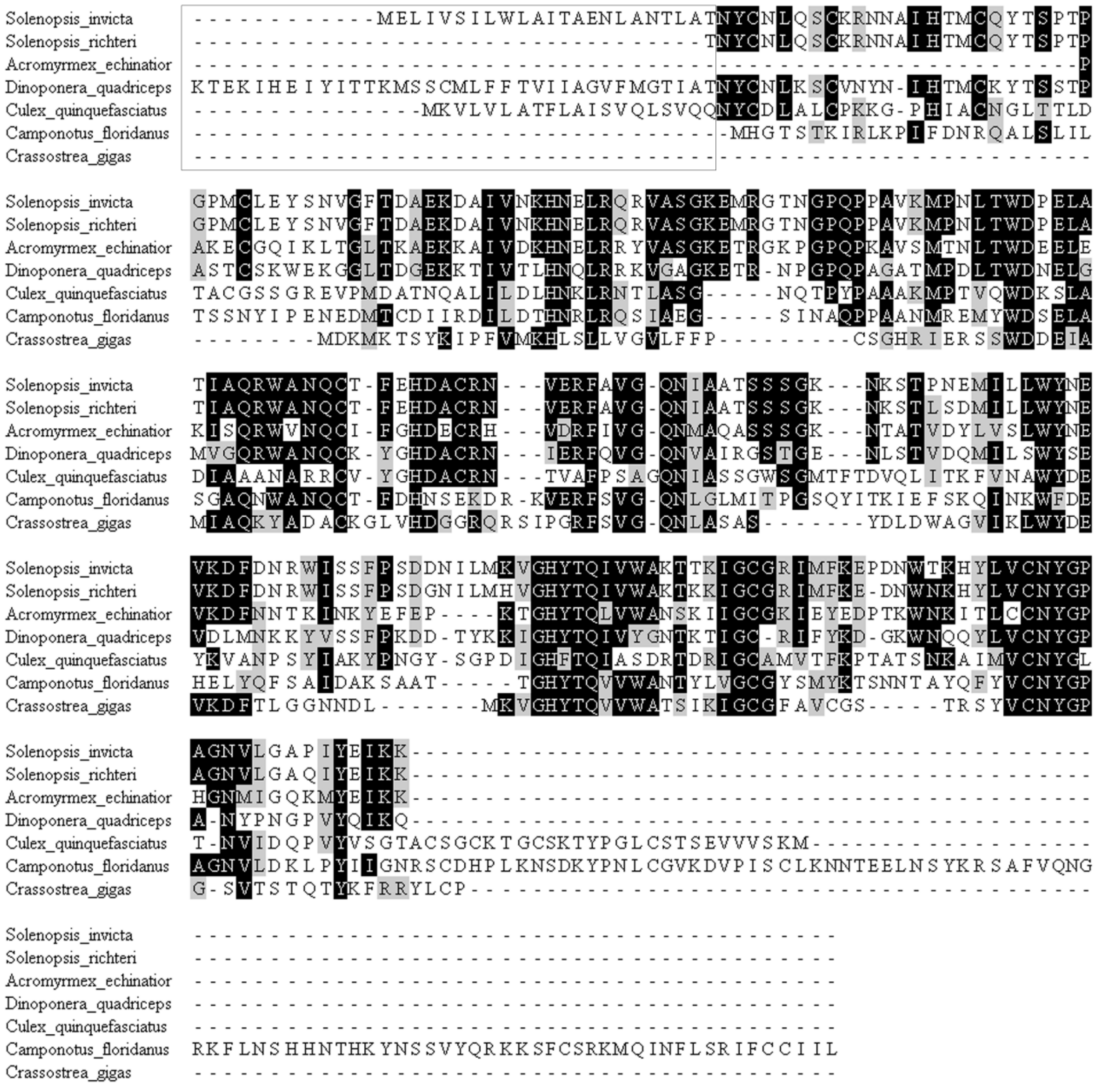
Multiple alignments of the deduced amino acid sequences of the venom allergen antigen 5 (Sol i 3) of *D. quadriceps* with known sequences from different Hymenoptera species. The conserved regions of the Sol i 3/allergen antigen 5 (Ag 5) polypeptide sequences of *D. quadriceps* and other hymenopterans are presented in black boxes. Conserved amino acid residue mutations are highlight in light gray boxes. Dots represent gaps. The unique amino acid sequences of the *D. quadriceps* allergen homologous to Sol i 3 protein are displayed in open boxes.

**Figure 8:**
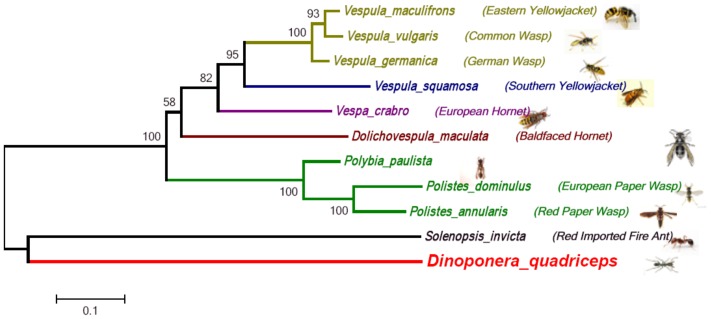
Comparison of Sol i 3/Allergen Antigen 5 polypeptides from *D. quadriceps* with their hymenopteran orthologs. Phylogenetic tree based on neighbor-joining analyses of a concatenated alignment of allergen 5/Sol i 3 and the orthology relationships of multiple insects. The scale bar indicates 0.05 substitutions per site. *Vespula maculifrons*, *Vespula vulgaris*, *Vespula germanica*, *Vespula squamosal*, *Vespa crabro*, *Dolichovespula maculate*, *Polybia paulista*, *Polistes dominulus*, *Polistes annularis*, and *Solenopsis invicta*.

**Figure 9:**
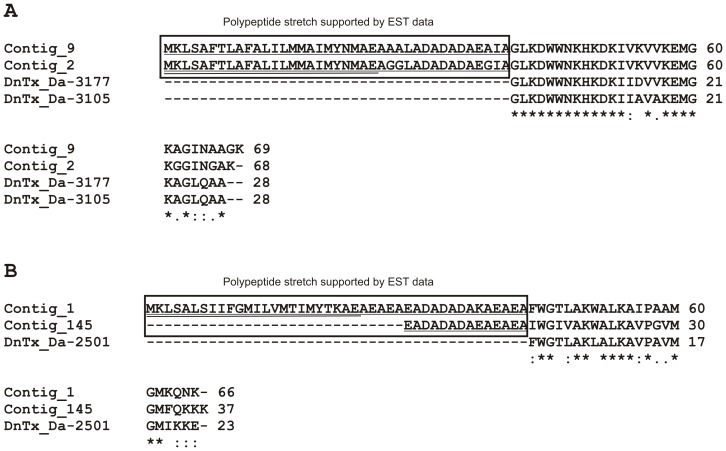
Multiple alignments of deduced amino acid sequences of different identified dinoponeratoxins from the *D. quadriceps* venom gland transcriptome and *D. australis*. Deduced *D. quadriceps* dinoponeratoxin cDNA precursor sequences (contig_1 and contig_9). RNA deep sequencing contigs (contig_2 and contig_145) were compared to mature peptide sequences from *D. australis* (Da-3177 and Da-3105) (part A) and from another species of *Dinoponera* (Da-2501) (part B). ClustalW was used to multi-align the sequences. Identical amino acid residues are marked with asterisks. Stretches of deduced amino acid sequences supported by EST sequences are boxed. Signal peptides (pre-peptides) are doubled underlined, and pro-regions of pro-peptides are shown with a single line under the sequence. Contigs 1 and 9 were first identified in the EST library, and contigs 2 and 145 came primarily from RNA-Seq.

From the RNA-sequencing data, Gene Ontology (GO) assignments were first used to classify the functions of these assembled transcripts. Based on sequence homology, 3024 transcript sequences were assigned at least one GO term, including 53 functional groups at the second level (Figure S2). Additionally, all assembled transcripts were compared against the Cluster of Orthologous Groups (COG) database to predict possible functions. The results revealed 1592 sequences with significant homology and assigned them into the appropriate clusters. These COG classifications were grouped into 23 functional categories (Figure S3, A to Z). Furthermore, according to the species and protein annotation information of the nr database, the transcripts related to toxins and venom components were grouped into the following four main categories: dinoponeratoxins, venom allergens, phospholipase-like toxin peptides and lethal-like proteins (Fig. 5). Again, as revealed by the cDNA library, there was a high preponderance of dinoponeratoxin transcripts. Venom phospholipase-related toxins and lethal-like proteins corresponded to the largest proportion of contigs. Specifically, the predominant encoded proteins found in the venom gland of *D. quadriceps* were the venom allergen 1 (Sol i 1) and venom allergen 3 (Sol i 3) antigens. Multiple alignments with sequences of other Hymenoptera species revealed several unique amino-acid sequence regions in Sol i 1 and Sol i 3 of *D. quadriceps* (Fig. 6, Fig. 7). The Sol i 3 protein sequences that was orthologous to those of other hymenoptera species, especially bees, were subjected to phylogenetic analyses (Fig. 8). As expected, the phylogenetic tree showed that *D. australis* was more closely related to *S. invicta* (the red imported fire ant) than other bee species. We found four contig sequences that coded for dinoponeratoxins and were homologous to the same toxin class of another ponerine ant species; i.e., *D. australis*. Multiple alignments of the peptide sequences from *D. quadriceps* and the *D. australis* dinoponeratoxins (Fig. 9) showed that these toxin peptides are highly conserved but also exhibited several amino acid substitutions that reflect the species-specific diversification of this toxin.

Contigs that were not related to any known sequences in the nr database accounted for 65% of the total transcripts. This high percentage of “no match” contigs was not surprising because the *E*-value used in the BLAST search was stringent to ensure the reliability of the alignment results. Additionally, the numbers of annotated sequences related to venom from other ant species that are available in the nr database are limited. Moreover, *de novo* RNA-Seq assembly is a complicated process and probably resulted in some sequencing errors, repetitive sequences and aberrant amplicons that brought several “acceptable” incorrect assembled sequence fragments together. Nevertheless, these “no-match” contigs might represent mRNA transcripts that encode novel genes. To further identify and characterize the contigs that had no match in the nr database, these contigs were subjected to a blastx search against the UniProtKB database (2012). This analysis resulted in 34 best hit contigs. For the other “no match” contigs, we performed a BLASTn search against 411 ESTs obtained from the Sanger sequencing of the cDNA library of the venomous gland of *D. quadriceps*, and six contigs were well mapped by numerous ESTs from the venom gland of *D. quadriceps*'s (Table S4). Additionally, ORF prediction was implemented with the OrfPredictor server, which was designed for ORF prediction and translation of batches of EST or cDNA sequences [31]; then, signal peptides were analyzed with PrediSi [32]. These seven contigs were novel candidate venom encoding transcripts in *D. quadriceps* because they were dissimilar proteins that did not match with any other sequences deposited the nr or UniProtKB databases.

### Clusters related to proteins involved in venom gland physiology

The results of deep RNA-sequencing showed that the venom gland transcriptome was composed of a large proportion of housekeeping genes, such as ribosomal proteins, cellular process-related proteins and hypothetical proteins, but the proportion of venom proteins was small (Fig. 3). Additionally, we identified two sex determination proteins in the venom transcriptome that could be used to predict the phylogenetic relationships of selected ant and bee species. Our phylogenetic analyses indicated that *H. saltator* likely shares the closest parental species with the giant ant *D. quadriceps* and suggests these species might have evolved from a common ancestor (Fig. S4).

### Inhibitor cystine knot- (ICK-) toxin transcripts in the venom gland of *D. quadriceps*

**Figure 10:**
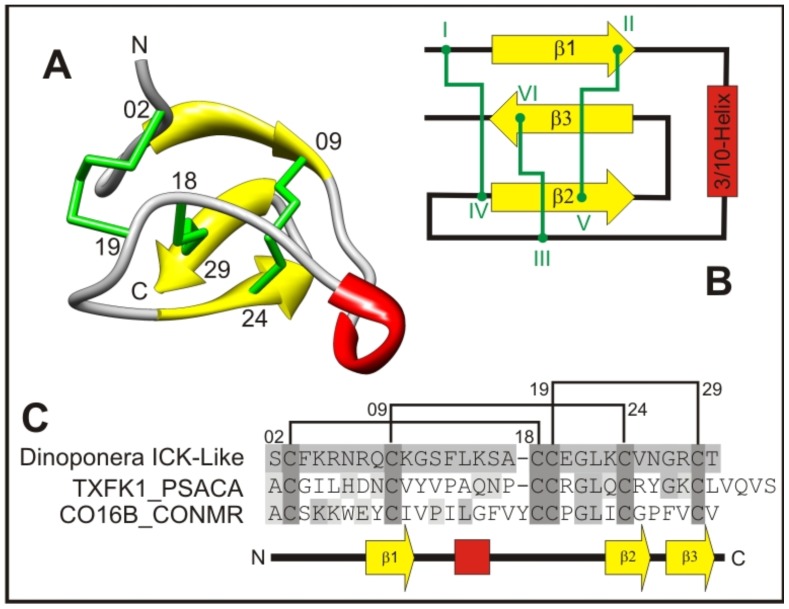
The Inhibitor cystine knot- (ICK-) toxin from *D. quadriceps* venom. A) *Dinoponera ICK-like* tridimensional homology model from contig 05 (Table S1) mature peptide (yellow – β-sheet; green – cysteine; ted – 3/10-helix. N-terminus (**N**); C-terminus (**C**). The numbers indicate the cysteine positions in the *Dinoponera* sequence. The presence of a ‘disulfide through disulfide knot’ structurally defines this peptide as a knottin. B) Schematic representation of a knottin obtained when one disulfide bridge crosses the macrocycle formed by two other disulfides, and the interconnecting backbone (disulfide III–VI) goes through disulfides I–IV and II–V. C) Alignments of the *Dinoponera ICK-like* mature peptide with the tarantula and Conus snail sequences of the templates used in the homology model showing the highly conserved cysteine framework [C-C-CC-C-C] that is characteristic of the omega-toxin-like family. **TXFK1_PSACA**: psalmopeotoxin I (PcFK1) from the venom of the tarantula *Psalmopoeus cambridgei* (NMR structure 1X5V; UNIPROT Accession P0C201). **CO16B_CONMR**: Mu-Conotoxin MrVIB from conus snail *Conus marmoreus* (NMR structure 1RMK; UNIPROT Accession Q26443).

A deduced amino acid sequence encoded by contig 05 (Table S1) has a putative signal peptide of 23 amino acids followed by a mature core toxin of 30 amino acids that includes six cysteine residues. A pro-piece (pro-peptide) was absent. As was inferred by similarity comparisons, the cysteine residues impair the structural configuration of the so-called “disulfide through disulfide knot” and allow for the grouping of this peptide into the class of knottins (Fig. 10-A and -B). The *Dinoponera* ICK-like toxin precursor contains a cysteine framework that is typified by VI/VII; i.e., -C-C-CC-C-C-, which is characteristic of the ω-toxin super-family (PFAM Clan CL0083) that encompasses several type-1 conotoxins, funnel web spider toxins and assassin bug toxins, among others. In figure 10-C, ICK-like toxins from *D. quadriceps*, *Conus* species, and spiders are aligned for primary structure comparison. Another type of ICK-folded toxin transcript found in the venom of the giant ant was similar to the so-called cysteine-rich venom proteins that share structural similarities with the toxins in the venoms of distinct species of animals (e.g., contig 1144, table S3).

## Discussion

Ants play significant ecological roles not only in diverse terrestrial environments, particularly in the tropical regions of the planet, but also in extreme biomes. Ants are engaged in several beneficial interrelationships with other organisms that include pollination, microbial dispersal, insect predation, and symbiotic associations with microorganisms [33]. In agriculture, ants modulate and interfere with soil conditions and may cause economic losses due to several factors including damage to the foliage of commercial plants, direct and indirect reductions in crop yields and pest management-related increases in the costs production [34]. With over 12,000 known species, the ants are among the most successful insects in the animal kingdom, and many of these species depend on venom for defense against other organisms, including predators and other ant species [35].

The genus *Dinoponera* inhabits the tropical regions of the New World, and its nests are located in diverse Brazilian biomes including semi-arid and savannah-like vegetation and humid and rain forests. *D. quadriceps* preys exclusively on insects and is normally harmless to humans except when they are disturbed or inadvertently encountered; the resultant interactions may result in accidents of varying levels of severity. Therefore, knowledge of venom composition is desirable not only to comprehend the pathophysiological basis of the effects and symptoms elicited by the sting of *D. quadriceps* but also to characterize proteins and peptides that will potentially be useful in the diagnosis and prospective treatment of chronic human diseases [5], [36]. The biotechnological applications of ant venom include not only the development of pharmaceuticals but also the development of bio-insecticides; the latter benefit is exemplified by poneratoxin, a small 25-residue neuropeptide that is active at the Na+- and K^+^-channels of invertebrates and was originally isolated in the venom of *Paroponera clavata* [29].

A robust strategy for the study of the venom profiles of small poisonous creatures with tiny venom glands relies on molecular cloning and DNA/RNA sequencing [37]. Thus, we prepared a *D. quadriceps* venom gland cDNA library and investigated the toxin repertoire of cloned transcribed genes by Sanger and RNA deep sequencing. Hundreds randomly picked clones from the Sanger sequencing are presented in figure 2 and table S1. Regarding the contigs and singlets datasets, proteins classified as “no hits” accounted for ∼20% of all ESTs, and dinoponeratoxins accounted for about 5% of all sequences categorized as toxins (Fig. 2-A). In terms of the absolute numbers of individual clones, over 37% of all retrieved random picked sequences were dinoponeratoxins (130 clones out 420) and over 50% of all venom-related sequences (Fig. 2-B). Two novel full-length dinoponeratoxin isoform precursors were identified in EST library (discussed below). Additionally, representative sequences of allergens and other minor components were also annotated and catalogued. However, due to the steep rise in the redundancy in the number of transcript sequences rescued from the cDNA (ESTs) library, we decided to conduct a deep investigation using next generation RNA-sequencing (NG RNA-Seq) to characterize the entire transcriptome of the *D. quadriceps* venom gland. To describe the overall transcript profile, we obtained our sequencing dataset with the Ion Torrent Personal Genome Machine (PGM™) and the Trinity program, which is specially designed for RNA-seq *de novo* assembly. This strategy yielded over 4,000,000 bp, and over 2,000,000 reads with an average length of ∼230 nucleotides were deep sequenced (Table S2). These reads generated over 18,500 contigs, of which 6,463 contigs corresponded to BLASTx hits. Most of the expressed sequences in the venom gland encoded proteins related to biological processes, cellular architecture, and molecular functions (Fig. 3 and Fig. S2). Importantly, based on functional classification, the most abundant proteins (over 200) were related to ribosomal structure and biogenesis, translational and post-translational modification, protein turnover and chaperones (Fig. S3), as it would be expected for a specialized and dynamic metabolic active tissue that needs to synthesize venom components for ant defense and feeding. Another interesting finding from the giant ant transcriptome is related to the proportion of Hymenoptera species with which *D. quadriceps* sequences share high similarity based on BLASTx queries. Nearly 60% of all *D. quadriceps* transcript sequences had the greatest homology with sequences from *H. saltator*, followed by *A. echinator* (∼11%), *S. invicta* (∼7%) and *M. rotundata* (∼6%) (Fig. 4). As *D. quadriceps* and *H. saltator* belong to the same subfamily (Ponerini) and evolved from a common ancestor, it is reasonable to argue that they have a common framework of transcript sequences in their venom glands. Two groups of highly homologous proteins expressed by hymenopterans in the venom gland transcriptome of *D. quadriceps* that were disclosed here are related to “sex determination” and mixed-function “lethal-like” proteins.

The so-called “sex determination proteins” were first discovered in *Drosophila* [38] and thereafter shown to be present in ant species, such as *A. echinatior, H. saltator* and *C. floridanus* (Figure S4). This group of proteins belongs to the BTB-ZF family of transcriptional regulators, which are part of the somatic sex determination hierarchy [39], [40]. These proteins can switch fruitless gene (fru) splicing from the male-specific pattern to the female-specific pattern through the activation of the female-specific fru 5′-splice site [41]–[42]. Our analysis of the molecular phylogeny of sex determination proteins indicated that the amino acid sequences of the members of this class of proteins are phylogenetically related to the insect clade and, conversely, that the orthologous relationship is closely related to insect species (Figure S4), which suggests a common mechanism of sex determination in hymenopterans. Moreover, the sex determination proteins are usually expressed in specific regions of the adult male brain that are associated with the control of courtship songs and the steps of male courtship and are also expressed in the larval and pupal male mushroom bodies and optic lobes and thereby possess tissue-specific characteristics [38], [39]. Importantly, our results have revealed for the first time that this group of proteins is expressed in giant ant venom glands. Further investigation is required to understand the role of the orthologous sex determination proteins in the venom gland, although the presence of this group of proteins might suggest that they influence courtship behavior and social organization in the queenless nests of the giant ant species.

Several classes of polypeptides with multiple functions are grouped together under the term “lethal-like proteins”, despite the fact that these proteins have distinct structures and protein motifs (Fig. 5 and Table S2). For example, we found transcript that coded for *protein lethal essential for life*, *multidrug resistance-associated protein lethal*, *lethal neighbor of tid protein*, and *lethal malignant brain tumor-like protein*, among other lethal-like sequences. In the category of *protein lethal essential for life*, we found sequences that possessed the signatures of small heat shock proteins (sHSPs) and **α**-crystallins (**α**C); i.e., domain sHSP/**α**C. Polypeptides with these domains, apart from being responsive to a variety of stresses, are involved in a wide range of functions in multiple cell types and multiple organisms [43]. In the venom glands of ants, the *protein lethal* with domain sHSP/**α**C proteins might work as co-chaperones that assist in toxin folding and post-translational processing of key proteins. The other lethal-like or lethal-related proteins, namely, the *lethal neighbor of tid proteins*, contain the typical domain of ALG3 proteins; i.e., a structural motif dedicated to the enzymatic linkage of N-glycosidic groups to glycoproteins (pfam05208). The catalytic process of glycosilation post-translationally modifies numerous toxins including ant venom phospholipases [44]. Lethal-like proteins are also associated with multidrug resistance. A number of transcripts code for *multidrug resistance-associated protein lethal* proteins, which include the ABC transporter cassette motif in their structures. In ants, these polypeptides might be associated with the active transport of glycoproteins and xenobiotics to sub-cellular compartments like those that have been observed in several organisms and described in more detail in *Drosophila* Malpighian tubule physiology [45], [46]. Another lethal-related protein class in the giant ant transcriptome includes the *lethal malignant brain tumor-like protein*. The members in this class of proteins contain the domain of the MBT superfamily (smart00561) as exemplified by proteins of *Drosophila* and vertebrates that act in the process of transcriptional regulation. Additionally, proteins in this class possess the “sterile alpha motif (SAM)”, which interacts with diverse proteins, RNAs and membrane lipids, and plays a role in signal transduction and transcription. Thus, the categorization of lethal-like protein in the venomics of the giant ant relies on the fact that members of this group of polypeptides participate in critical biochemical events of venom gland physiology and might additionally induce disruption of the cellular circuitries of prey or victims after ant sting and envenomation.

Notably, less than 1% (exactly 0.72%) of all sequenced transcripts corresponded to toxins or venom-related peptides in hymenopterans (Fig. 3), and this percentage corresponded with hundreds of polypeptides. Accordingly, Johnson and colleagues [30] performed a proteomic-based investigation on the venom of *D. australis* and found over 75 polypeptides, but only described the six most abundant dinoponeratoxins and their congeners. In another recent study on the venom transcript profile of ants, Bouzid and collaborators [2] identified five major classes of toxins and toxin-related peptides (i.e., pilosulin 5-like, insect cytokine precursor, Sol I 3 antigen, secapins and allergen peptide) that corresponded to a total of 42 of the 85 ESTs that were Hymenoptera toxin-like candidates.

Despite of relative simplicity of ant venoms, which are comprised of a basic framework of allergen peptides, hyaluronidases, phospholipases and phosphatases, the small repertoire of venom-related components is highly effective in causing tissue permeability, edema, warmth of the local area, life-threatening systemic anaphylactic reactions, and, occasionally, delayed hypersensitivity; these reactions are observed after most Hymenoptera stings [47]. Moreover, due to the high levels of structural conservation and homology among hymenopterans allergens, venom cross-reactivity is one of the major risk factors related to insect stings [48].

In our transcriptome analysis, we determined that the giant ant venom gland possesses a relative simple toxin and venom-related peptide repertoire, the sequences of which predominantly fit into the following three major venomic classes: esterases (phospholipase-related polypeptides and carboxylesterases), venom allergens, and dinoponeratoxins (Fig. 2-B and Fig. 5; table S1 and S3). Minor venom components, which are not less toxic or harmless to human cells and tissue, included cysteine-rich peptides stabilized by disulfide bonds that are distributed across the venoms from animals of multiple phyla.

In table 2, the general venom polypeptide cores from *D. quadriceps* and several species of ants are shown and compared. There are venom components common to all species (e.g., phospholipases) and others, such as venom allergens and dinoponeratoxin, which are genus-specific. However, given that most of the common venom components share high levels of amino acid and epitope similarity, cross reactivity and anaphylaxis after Hymenoptera stings is a recurrent risk, as previously mentioned. This is a particular concern for phospholipases and other major venom allergens, which exhibit the highest levels of similarities in their primary structures and tri-dimensional topologies.

**Table 2:** Overview of major venom polypeptide components of *D. quadriceps* and other ant species.

Subfamily	Genus and species	Major (core) venom polypeptides	Reference
**Ponerinae**	*Dinoponera quadriceps*	Dinoponeratoxins, allergens (homologous to Sol i 1/PLA_1_B, Sol i 2/4 and Sol i 3/Ag 5), esterases (phospholipases A and B, acid phosphatases, carboxylesterase)	This work
	*Pachycondyla sp.*	16 and 24 kDa (Pac c 3/Sol i 3 allergen) IgE-binding proteins	[44]
	*Harpegnathos saltator*	Esterases	[75]
**Myrmicinae**	*Solenopsis invicta*	Allergens (Sol i 1, 2, 3 and 4), phospholipases (including Sol i 1/PLA_1_B)	[44]
	*Tetramorium bicarinatum*	Pilosulin-like peptides (1,3, 5), allergen (Sol i 3/Ag 5), insect cytokine precursor uENF2, metalloproteases	[2]
	*Acromyrmex echinatior*	Carboxylesterase-3, Esterase FE4, Phospholipase A1,	GenBank GI68854, GI68747
**Formicinae**	*Camponotus floridanus*	Carboxylesterase-3	[75]
**Myrmeciinae**	*Myrmecia sp.*	Phospholipase A_2_, Phospholipase B, Hyaluronidase, Acid and Alkaline phosphatases	[92]

Notably, two of the most conserved venom allergens from fire ants (*S. invicta*) are also expressed in the venom gland of *D. quadriceps*: the homologous of Sol i 1 and Sol i 3 (Fig. 6 and Fig. 7, respectively). *D. quadriceps* venom allergen 1 belongs to the phospholipase AB_1_ group and, consequently, displays a propensity for cross-reactivity with the Sol i 1 homologs of several hymenopteran-related sequences [49]. Allergen proteins are potent components of the Hymenoptera venom arsenal that induce hypersensitive allergic responses. As can be observed in figures 7 and S5, the structural conservation of the *D. quadriceps* Sol i 3/allergen 5-like transcript is not only related to sequences from other species of ants but also to sequences from wasp species. Indeed, these sequences are collectively called CAP proteins (**c**ysteine-rich secretory proteins - CRISPs, insect venom **a**llergen antigen 5, and **p**athogenesis-related 1 proteins), and they compose a superfamily of polypeptide sequences that are found in a wide range of organisms, including prokaryotes. In figure S5, the *D. quadriceps* CAP allergen Sol i 3/allergen 5-like is compared with homologous sequences from *S. invicta* and *Vespula vulgaris*, and its tri-dimensional model depicted. The spatial structure of *D. quadriceps* Sol i 3/allergen 5-like proteins extremely highly conserved and fits well with fire ant Sol i 3/Ag 5 experimental crystal structure data [50]. Interestingly, polypeptides in the mammalian male reproductive tract and in the venom secretory ducts (e.g., stecrisp, PSTx, triflin, helothermine) of several snake and lizard species are CAP proteins in the CRISP group. CRISPs have two domains: A CAP N-terminal domain followed by a C-terminal cysteine rich domain (CRD) containing 10 absolutely conserved cysteine residues. Importantly, from the perspective of venomics, the CRD possesses a fold in common with sea anemone ion channel inhibitors (Bgk- and Shk-type toxins), which are involved in the blocking of voltage gated Ca^2+^- and K^+^-channels and modulate ryanodine receptors. Interestingly, these biological activities are only observed when these polypeptides are associated with the CAP domain [51], [52]. Despite belonging to CAP superfamily, the Sol i 3/allergen antigen 5 (Sol i 3/Ag 5) homologs from *Hymenoptera* venom form a major and distinct clade. Sol i 3/Ag 5 homologs, besides having been initially characterized in the venoms of fire ants and several species of wasps, have also been identified in the midgut of *Drosophila* and in the saliva of ticks, sand flies, and mosquitoes. In the secretions of blood-feeding insects, the Ag5 proteins are part of a cocktail of salivary proteins that are believed to be active either in the suppression of the host immune system or in the prevention of clotting to prolong feeding [52]. Thus, the structural conservation of these allergens in the venom of *D. quadriceps* (Ponerinae), other ant species (e.g., *Pachycondyla* and *S. invicta*) and wasps is an interesting aspect of Hymenoptera venomics because these insect groups have a long natural history of divergence that dates to more than 150 million years ago [35].

In addition to the venom allergen homologous of Sol i 1, which are related to the group of phospholipase AB_1_,esterases (phospholipases and carboxylesterases) comprises another class of predominantly expressed venom components in the transcriptome of the giant ant *D. quadriceps*. We found members and isoforms of PLA_1_, PLA_2_, PLB_1_, PLD, which indicates that phospholipases compose an important part of the toxin core of giant ant venoms (Fig. 5, table S3). Broadly, the class of esterase-lipases comprises a superfamily of enzymes that includes diverse groups of phospholipases (A1, A2, B, C and D). The most well-known phospholipases, A_1_, A_2_ and C (PLA_1_, PLA_2_ and PLC), hydrolyze the ester bonds of phospholipids at the specific positions of *sn-1*, *sn-2* and *sn-3*, respectively [53]–[55], and the resulting products are used as precursors in the synthetic pathways for pharmacological mediators such as leukotrienes and prostaglandins or play a role in signal transduction as secondary messengers [56]. PLA_1_s can be classified into two groups based on their structures and cellular localizations. The first group encompasses intracellular enzymes that include phosphatidic acid-preferring phospholipase A_1_, and the second group is composed of other extracellular enzymes that include phosphatidylserine-specific PLA_1_ [57]. Members of the latter group of enzymes have been detected in hymenopterans, mainly in wasp species. The structures of these enzymes are conserved across a wide range of organisms, and, in humans, they induce platelet activation, allergic reactions and hemolysis [54], [58], [59]. Phospholipases A_2_ is subdivided into more than a dozen subtypes (from I to XV, with subgroups) and is the most abundantly expressed type of phospholipase in the venom of hymenopterans. The venoms of bees and wasps have been shown to possess high levels of phospholipase activity; thus, this class of enzymes has been implicated in tissue damage and hypersensitivity reactions [60]–[62]. The pioneering report of Schmidt and Blum [63] detailed the high phospholipase A_2_ and B contents of the venom of the Harvester ant (*Pogonomyrmex badius*). Since that early work, phospholipase activity has been described in the venoms of several ant species, including *Neoponera apicalis*, *N. olivaceae, P. clavata, Pachycondyla cressiondes, Pogonomyrmex occidentalis* and *Dinoponera grandis* [53]. These enzymes have been related to be the major allergen in hymenoptera venoms [49], [64], [65] and, notably, are responsible for the antigenic cross-reactivity between hymenoptera venoms [49], [60], [66], [67].

PLCs are ubiquitous enzymes that hydrolyze membrane lipid phosphoinositides to yield two important second messengers, inositol phosphates and diacylglycerol (DAG) (cl14615); however, despite finding numerous types of phospholipases, we did not find any PLC venom in our study of giant ant venomics, Two additional subfamilies of phospholipases should be mentioned: (1) phospholipase B, which has both esterase and phospholipase-A/lysophospholipase activity (cd01824) and is involved in the conversion of phosphatidylcholine to fatty acids and glycerophosphocholine (perhaps in the context of dietary lipid uptake); and (2) phospholipase D (PLD), which is a signal-activated enzyme that catalyzes the hydrolysis of the phosphodiester bond of glycerophospholipids to generate phosphatidic acid (PA); PA is an intracellular signaling lipid that has been implicated in vesicular trafficking [68], [69]. In the *D. quadriceps* venome, we found transcripts for the first giant ant PLD (sphingomyelinase-D, E.C. 3.1.4.41), a dermonecrotic toxin that, until now, had only been described venoms of spiders, particularly the brown spider *Loxoceles gaucho*. In spider venom, PLD is responsible for the necrotic arachnidism and massive inflammatory response observed in loxoscelism, which is the main effect of brown spider envenomation [70], [71]. Thus, the presence of PLD in the *D. quadriceps* venome is another remarkable finding that advances the comprehension of the pathophysiological effects of the venom of the giant ant. Moreover, the expression of several isoforms and subgroups of phospholipases in the giant ant venom gland indicate that lipid homeostasis and the lipid-mediated signaling circuitry are targets that mediate the tissue disruption of, and damage to, prey and/or victims.

We also identified transcripts for carboxylesterases in the venom of *D. quadriceps*; carboxylesterases are enzymes that hydrolyze carboxylic acid esters into their corresponding acids and alcohols (GO0004091). Enzymes in this class are considered to be protective molecules because promote cellular detoxification by inactivating toxicants and carcinogens. As described by Hatfield and Potter [72], diverse molecules such as drugs, pesticides and veterinary products contain ester moieties that are susceptible to catalytic conversion by these enzymes. Interestingly, carboxylesterases have been identified in diverse hymenoptera venoms, including the venoms of *Apis mellifera* [73], *N. vitripennis* [74], *H. saltator*, *C. floridanus* [75], *A. echinatior* (GI332028825). Thus, it is likely that such this class of venom components might be involved in the detoxification xenobiotics and confer resistance to insecticides [74], [75]. Despite this physiological function, the *A. mellifera* carboxylesterase has been shown to cause allergic reactions in humans [76], and this type of venom component might make a similar contribution to the symptoms of by giant ant stings.

Dinoponeratoxin transcripts were identified in high proportions in the transcriptome of *D. quadriceps*, and four novel dinoponeratoxin sequences were discovered (Fig. 9). As noted by Johnson and co-workers [30], dinoponeratoxins share variable primary sequence similarities with antimicrobial peptides from ponerine ants (i.e., ponericin G and W3) and other organisms; e.g., frogs (gaegurin-5 and brevinin-1 PTa). Additionally, it is notable that bioactive peptides seem to be encrypted into the dinoponeratoxin precursors, as numerous peptide fragments were detected in the venom proteomic of *D. australis*. The alignment of dinoponeratoxins with antimicrobial peptide sequences provides a clue regarding the functions of these peptides in the venom of *D. quadriceps* and other ponerine ants, although the actual biological activity of dinoponeratoxin - other than microbicide - deserves further investigation. It is known that many antimicrobial peptides cause cellular membrane damage via pore formation or micellization as a part of their modes of action [77], [78]; these processes might intensify toxin dispersal in the tissues of prey or victims and increase the efficacy of ant envenomation. Another interesting aspect of dinoponeratoxin transcripts is related to their cDNA precursors at the nucleotide level. We observed that the dinoponeratoxin (contig 9, table S1) precursor shares a certain degree of identity with the 5′-end of the pilosulin 5 cDNA precursor (not shown). First discovered in the venom of Australian jumper jack ant (*Myrmecia pilosula*), pilosulins comprise a family of short monomeric and dimeric peptides with antimicrobial activity that is mediated through histamine-releasing and hemolytic effects [79], [80]. In light of toxin evolution and diversification, the sharing of stretches of nucleotide sequences between dinoponeratoxin and pilosulin-5 is indicative of the conservation of parental ancestor genes and their recruitment as a template for the evolution of bioactive peptide variants in the venom that, in this case, cause the degranulation of mast cells and histamine release. Another pilosulin-related sequence of giant ant venom gland was observed to be encoded by the contig 6 (table S1).

Minor toxin-related components identified in the venom gland transcriptome of *D. quadriceps* include sequences with high homologies with the spider U8 agatoxin, the venom protein VN50, the cysteine-rich venom proteins of the endoparasitic wasp, and the ICK-like toxins from *Conus* and spiders. The U8-agatoxin-Ao1a-like isoform peptide precursor was found in an initiative to identify toxin-like structures based on new algorithms for the interrogation of the cDNA library of the spider *Agelena orientalis* and the data bank for neurotoxin sequences [81]. Based on this strategy, a contig (1144_B2-2, table S3) from the *D quadriceps* transcriptome was found to meet the criteria for the “extra structural motif” (ESM), in which a pair of CXC fragments in the C-region (or C1CZC1C, where Z is variable number of amino acid residues), code for a novel neurotoxin precursor that is homologous to spider toxin and diverse hymenopteran-related sequences (Fig. S6).

The venom protein VN50 is a serine protease homolog without enzymatic activity from the venom *Cotesia rubecula* that, once injected into the host, interferes with the immune defense mechanisms mediated by the prophenoloxidase (proPO) activation cascade [82]. The presence of a VN50-related sequence (contig 1219_B2-2) in the venom gland of *D. quadriceps* indicates that this venom component has the ability to disturb the homeostasis of invertebrates and augment the processes of their intoxication. Another less abundant, but not less important, transcript sequence in the *D. quadriceps* transcriptome (contig 23_B2-2) shares high similarity with the cysteine-rich venom proteins that make up a significant fraction of the polypeptides from the venom of the parasitoid wasp *Pimpla hypochondriaca* [83]. Venom peptides in this class include trypsin inhibitors (including the Kunitz type) and voltage-gated Ca^2+^-channel antagonists (ω-conotoxins) with analgesic properties from the gastropod mollusk sea snail *Conus*. A structural fold that is highly conserved across diverse bioactive venom cysteine-rich peptides (or disulfide-rich domains) is known as the inhibitor cysteine-knot (ICK) motif. The ICK motif is an evolutionarily conserved structure that is found in the venoms of a wide range of organisms of diverse phyla, including arthropods (spiders and scorpions), marine cnidarians (sea anemones), sea snails, fungi, plants, and vertebrates. The ICK motif is a chemically and thermally very stable protein fold that is characterized by a knot formed by segments (α-helix, β-sheet or random coil) connected by two disulfide bonds and interlaced with a third disulfide bond that forms up to six loops with variable sequences [84]. Venom from the marine mollusk *Conus*, spiders and scorpions include numerous toxins containing the ICK fold. In the transcriptome of *D. quadriceps*, we identified the first ponerine ant ICK-containing toxin (Fig. 10). The BLAST hits with the highest scores for this novel sequence were for the superfamily-A conotoxins and the scorpion like-toxins. The high scores between this novel sequence and members of these protein families were mainly due to similarities with the signal peptides of the *Dinoponera* ICK-like toxin precursors. However, the *Dinoponera* ICK-like toxin cysteine framework was proven to be VI/VII (C-C-CC-C-C), and this pattern of disulfide bonds would place this ant ICK-like toxin into the ω-toxin superfamily (PFAM Clan cl0083). This superfamily includes several type-1 conotoxins, funnel web spider toxins, and assassin bug toxins, all of which are characterized as neurotoxic antagonists of several subtypes of ion channel [85]. Curiously, the translated sequence of the *Dinoponera* ICK-like toxin also has structural homology with an uncharacterized protein family expressed that is in *Schistosoma* (result not shown). Moreover, ICK-type peptides with antimicrobial have recently been identified in the genomes of seven ant species; i.e., *A. cephalotes*, *A. echinatior*, *L. humile*, *S. invicta*, *P. barbatus*, *C. floridanus*, and *H. saltator*. Based on molecular phylogenetic analyses, these ant antimicrobial peptides with ICK motifs have been were categorized into three subtypes [86]. Thus far, transcripts encoding cysteine-rich peptides with variable numbers of disulfide bonds and cysteine stabilized ICK-folded peptides form a minor proportion of all expressed components of *D. quadriceps* venom.

### Conclusions

In this study, we sequenced and characterized the transcriptome of the *D. quadriceps* venom gland and focused principally on the polypeptide core of the venom that is responsible for the symptoms caused by giant ant stings. With a combination of Sanger sequencing of the cDNA library and next generation deep RNA sequencing, a complete description of transcriptome of the venom gland was achieved. These experiments revealed the expression of coding sequences dedicated to specialized tissue physiology and toxin production. The polypeptide component framework of the venom is comprised of a major and a minor core of toxin classes, which, in combination, elicit local and systemic reactions to stings from this species of ponerine ant. The major toxin core is composed of venom allergens, phospholipases, carboxylesterases, lethal-like toxins, and dinoponeratoxins. With exception of dinoponeratoxins, all components of the major toxin core of the giant ant display high structural similarities with toxins of the same groups of proteins from different hymenopterans species (ants and wasps). This finding indicates that cross-reactivity is a recurrent phenomenon of hymenopteran envenomation and a property of the structures of venom components that has been maintained over the course of more than 100 million years of insect evolution and diversification. Moreover, the toxins that compose the major core may functionally interfere with lipid homeostasis and signaling, the trafficking of vesicles and protein-protein and protein-nucleic acid interactions. These toxins may also disrupt the biochemical circuitry of the tissues of the prey or victims. Dinoponeratoxins are another major toxic component of *D. quadriceps* venom and are exclusively expressed in the giant ant venom. This group of small disulfide-less toxins shares structural similarity with sequences from *D. australis* and, at the nucleotide level, shares an evolutionary relationship with pilosulins, which are antimicrobial and mast cell degranulating peptides from *Myrmecia* venom. Cysteine rich polypeptides that are shaped by disulfide bonds and folded into the typical ICK-motif comprise a fraction of the minor *D. quadriceps* venom protein core. The ICK-type peptides from the giant ant possess structural identity with peptides from scorpions, spiders and the marine venomous *Conus* gastropods. A component of the minor venom protein core of *D. quadriceps* is the homolog of a serine protease that is devoid of catalytic activity (i.e., the peptidase S1-derived scaffold venom protein of the solitary wasp and blood-feeding bat), which should interfere with innate immune responses and inhibit endogenous peptidase S1 homologs of the prey or victims. Taken together, the results of the transcriptome and venomic analysis of *D. quadriceps* provide an inestimable resource for the understanding of the molecular and physiopathological bases of giant ant envenomation and basic knowledge about the major and minor polypeptide venom components that may be useful in the future for medical biotechnology.

## Methods

### *Dinoponera quadriceps* sampling and processing

Specimens of the giant ant *Dinoponera quadriceps* were collected at twilight in the Serra de Maranguape (3°52′51″S and 38°42′39″W), which is a mountain range with peaks below 1000 m that is located about 40 km from the coast of the north-eastern state of Ceará, Brazil. A trap made with ground tuna meat was used to attract ants from inside their nests. The individuals were either kept in the laboratory in artificial nests or dissected with thin scissors and scalpels under a stereomicroscope (Nikon SMZ 800, Tokyo, Japan). The resting (not milked) venom gland was carefully separated from the other surrounding tissues, such as the Malpighi tubules and muscles, and quickly transferred to microtubes containing RNAlater solution (Life Technologies, U.S.A.). Authorization (no. 28794-1) to access Brazilian biodiversity was issued by the Brazilian Institute of the Environment and Renewable Natural Resources (Instituto Brasileiro do Meio Ambiente e dos Recursos Naturais Renováveis, IBAMA) of the Ministry of the Environment of the Federal Government of Brazil.

### RNA isolation and venom gland cDNA library construction

Total RNA was prepared using the RNAeasy Mini Kit (Qiagen, U.S.A), which is based on a combination of guanidine-isothiocyanate lysis and silica-membrane purification in a mini-spin column. The procedures were performed according to the manufacturer's instructions. The quality of the RNA was analyzed with denaturating RNA electrophoresis in TAE agarose gel as described in [87] and quantified by UV absorption using NanoDrop (Thermo Scientific, Wilmington, DE-USA).

The cDNA library was constructed using a switching mechanism at the 5′ end of the RNA template to preferentially amplify the poly-A tail (IN-FUSION™ SMARTer™ cDNA Library Construction kit, Clontech, Mountain View, CA-USA). Briefly, the synthesis of single strand cDNA began with 1 µg of total RNA and 3′ In-fusion SMARTer CDS primers. The resulting first strands of cDNA were sequentially amplified with SMARTer V Primer. To synthesize double strand cDNA, the Advantage 2 Polymerase, the 5′ PCR primer IIA and 3′ In-fusion SMARTer PCR Primer with the lowest possible number of cycles (in this case 15) were used. The double stranded (ds-) cDNA was fractionated by size exclusion chromatography (CHROMA SPIN 400, Clontech, USA), and the fractions with specific molecular sizes (over 400 bp) were combined and precipitated with NaCl/ethanol. The cDNA was quantified by U.V. spectrophotometry and ligated into the plasmid vector (pSMART 2IFD). A commercial strain of *E. Coli* was used to as a host for the maintenance and propagation of the *D. quadriceps* venom gland cDNA library, which was then stored at −80°C.

### Sanger sequencing of independent clones

The cDNA library was seeded in LB agar plates supplemented with appropriate antibiotics, and individual clones were selected and analyzed for insert size. To screen for positive clones, specific primers flanking the multi-cloning site of the vector were used in a PCR reaction. The amplified screening products were analyzed in 1% agarose gel and photodocumented. The PCR products of 420 independent clones were cleaned up by EXO/SAP treatment and sodium acetate/ethanol precipitation. The precipitated DNA were resuspended in deionized formamide and sequenced in a 3100 Avant Sequencer (Applied Biosystems, USA) using Prism Big Dye Terminator v3.0 Cycle Sequencing Ready Reactions (Applied Biosystems, USA).

### Data analysis of Sanger sequencing

Sequences were analyzed with the CLC Bio Main Workbench v 6.8.3 Software. The sequences were trimmed to exclude vector contamination and the poly-A tail. The following parameters were selected for clustering and assembling: minimum aligned read length of 50 and medium (ambiguity level) alignment stringency. The resulting contigs (clusters and singlets) were compared against the non-redundant GenBank (National Center for Biotechnology Information, http://www.ncbi.nlm.nih.gov) using BLASTx for all organisms and/or an arthropod databank (see below for deep sequencing analysis).

### Sample preparation and deep Sequencing using Ion Torrent Technology

A fragment library was generated with an input of 100 ng DNA using the Ion Xpress Plus Fragment Library Kit (Life Technologies, Carlsbad, USA). Next, DNA fragmentation, adaptor ligation, fragment size selection and library amplification were carried out for a total of 8 cycles according to manufacturer's instructions. The targeting of fragments of approximately 330 bp was performed with 2% agarose gel cassettes for the Pippin Prep instrument (Sage Science, Beverly, USA). Prior to emulsion PCR, the size distribution and concentrations of the library were assessed with a Bioanalyzer 2100 using the DNA High Sensitivity Kit (Agilent, Santa Clara, CA). The fragment library was adjusted to approximately 26 pM and amplified with Ion Sphere particles™ (ISPs) by emulsion PCR using the Ion OneTouch™ Instrument with the Ion OneTouch™ 200 Template Kit (Life Technologies, Grand Island, NY) and template-positive ISP enrichment according to the manufacturer's protocol (Part Number 4472430 Rev. E, 06/2012). Over 50% of the ISPs were and then sequenced on an Ion 316™ chip using the Ion Torrent Personal Genome Machine (PGM™) (Life Technologies, San Francisco, CA, USA) for 130 cycles (520 flows) with the Ion PGM™ 200 Sequencing Kit (Part Number 4474246 Rev. D, 06/2012).

### Sequence filtering and (*de novo*) assembly of deep sequencing data

Ion Torrent Personal Genome Machine (PGMTM) sequencing can generate 200–500 base pairs (bps) from each single end (SE) of a cDNA fragment. The raw reads were initially processed to obtain clean reads by removing the adapter sequences and removing low quality sequences (i.e., reads in which more than 50% of the bases had quality value s≤5). Next, de novo transcriptome assembly was carried out using the short-read assembling program Trinity [88]. Specifically the k-mer counting method was set to jellyfish, and the contigs that could not be extended on either end were defined as transcripts.

### Gene ontology and functional annotation

All of the assembled transcripts were searched against the NCBI non-redundant protein sequence (nr) and COG databases using BLASTX to identify the proteins with the highest sequence similarities with the given transcripts to retrieve the function annotations of the transcripts, and standard cut-off E-value of <10^−5^ was used. Next, the species and function information from the nr database was used to perform taxonomic analyses with in-house Perl scripts. For the nr annotation, the in-house BLAST2GO embedded annotation pipeline was used to obtain the GO annotations of the assembled transcripts to describe the biological processes, molecular functions and cellular components of the transcripts [89], and the WEGO [90] software was used to conduct GO functional classification to understand the distribution of gene functions at the macroscopic level. Furthermore, the transcripts that had no matches in the nr database were subjected to another BLASTX search against the UniProtKB database.

### Bioinformatic analyses of similarities and molecular phylogenies

For particular transcript sequences, systematically similarity analyses were performed using the BLASTP tool against the non-redundant (nr) and UniProt databases, and these analyses were subsequently restricted to the *Arthropoda*, *Hymenoptera* or *Formicidae* taxa. Further, the analyses were expanded to include more specific databases such as ArachnoServer, Knottins [91] and SCOP KNOTTIN Superfamily. Predicted amino acid sequences were aligned with ClustalW software using the default parameters (http://www.ebi.ac.uk), and the putative cleavage sites related to signal peptides (and pro-peptides) were deduced and compared with the available experimental crystal structure or proteomic data

## Supporting Information

Table S1 **Annotations of identified ESTs retrieved from randomly selected clones in the** ***D. quadriceps*** **venom gland cDNA library.** Contigs assembled from Sanger sequencing data according to described in the ‘materials and methods’ section, The Uniprot and sequence identification (GI) numbers of homologous polypeptides are indicated. Number in parenthesis represents percentage of sequence similarity.
(DOC)

Table S2 **Overview of experimental data from the next generation RNA sequencing.**
(DOC)

Table S3 **Annotations of the identified venomous contigs in the** ***D. quadriceps*** **venom gland.** Contigs representing candidate toxin-like peptides based on the alignment of EST data set, prediction of signal peptides and ORFs, and BLASTx comparison. GI denotes sequence identification number of “top hit” toxin homologous. Number in parenthesis represents percentage of sequence similarity.
(DOC)

Table S4 **Summary of toxin-like peptide candidates from the** ***D. quadriceps*** **venom gland transcriptome.** Validation of some RNA-Seq assembled contigs according to the mapped EST fragments. Full-length open reading frames (ORFs) are indicated with a “+” sign. The presence or absence of a signal peptide in the ORFs is indicated by a “Y” (yes) or “N” (no).
(DOC)

Figure S1 **The length distribution of the assembled transcripts of** ***D. quadriceps*** **venom gland.** Sequence length is expressed as base pairs (bp), and the frequency is expressed as the absolute number of sequences. The y-axis indicates the number of sequences of different lengths.
(TIF)

Figure S2 **Gene ontology (GO) classification of contigs identified in the venom gland of** ***D. quadriceps*** **.** Distribution of unigenes associated with biological processes, cellular components and molecular functions of the giant ant venome transcripts.
(TIF)

Figure S3 **Clusters of orthologous groups (COG) function classification of the assembled giant ant venomic transcripts.** Query of the COG database allowed for the classification of venom-related transcripts into 24 group based on functions (from A to Z) and attributes. The distribution of these groups of the *D. quadriceps* transcriptome (y-axis) is shown.
(TIF)

Figure S4 **Comparison of sex determination proteins from** ***D. quadriceps*** **with their hymenopterans orthologs.** Phylogenetic tree based on neighbor-joining analyses of a concatenated alignment of a sex determination protein and orthology relationships in multiple insects. The scale bar indicates 0.05 substitution per site. *Apis mellifera, Megachile rotundata, Bombus impatiens, Nasonia vitripennis, Acromyrmex echinatior, Harpegnathos saltator, Solenopsis invicta, camponotus floridanus*.
(TIF)

Figure S5 **Structural comparison of** ***D. quadriceps*** **CAP venom allergen antigen 5 with two hymenopteran venom allergen and the tridimensional topology of CAP venom allergen antigen 5.** The *Dinoponera* venom allergen precursor was aligned with the following Hymenoptera allergen Ag5 precursors using the BLOSUM62 scores: VA3_SOLIN (*Solenopsis invicta* venom allergen 3, Sol I 3) and VA5_VESVU (*Vespula vulgaris* Venom allergen 5, Ves5). Part A depicts the primary sequence analysis. The signal peptide is indicated by an orange box; the predicted pattern of disulfide bridges is represented by connected lines and numbered based on *Dinoponera* CAP Venom allergen 5 (dark gray box). The major conserved secondary structural elements are indicated by marks above the relevant amino acid sequences (red bar, α-helix; yellow arrow, β-strand). CAP signature motifs are marked by cyan boxes, and the histidine and glutamine/glutamic acid residues involved in the putative active site are also indicated (blue*). The histidines, which have the ability to form complexes with divalent cations, form a structure with some similarity to the protease active sites, which is consistent with the calcium-activated serine protease-like activity that has been reported for the *Conus textile* cysteine-rich venom protein endoproteinase Tex31 (Q7YT83). Part B: tri-dimensional structure based the on structural characteristics described in part A.
(TIF)

Figure S6 ***D. quadriceps*** **U8-agatoxin-Ao1a-like venom homolog Contig1144_B2-2 was assembled with data from the RNA-Seq reads.** TXAG8 (Q5Y4U4) is a U8-agatoxin-Ao1a-like venom homolog that was discovered by a program for the identification of toxin-related candidates in arthropod databases [81]. The other sequences came from query of the database for homologous entries.
(TIF)
