# Supplementary material for: Transcriptome Analysis in Venom Gland of the Predatory Giant Ant Dinoponera quadriceps: Insights into the Polypeptide Toxin Arsenal of Hymenopterans
Source: PLoS One. 2014 Jan 31;9(1):e87556. doi: 10.1371/journal.pone.0087556 (PMC3909188; doi:10.1371/journal.pone.0087556)
Supplement: Table S1 — Annotations of identified ESTs retrieved from randomly selected clones in the D. quadriceps venom gland cDNA library. Contigs assembled from Sanger sequencing data according to described in the ‘materials and methods’ section, The Uniprot and sequence identification (GI) numbers of homologous polypeptides are indicated. Number in parenthesis represents percentage of sequence similarity. (DOC) [file pone.0087556.s001.doc]

**Additional files**

**Table S1 – Annotation of identified ESTs retrieved from randomly picked clones in the *D. quadriceps* venom gland cDNA library.**

Contigs assembled from Sanger sequencing data according to described in the ‘materials and methods’ section. The Uniprot and sequence identification (GI) numbers of homologous polypeptides are indicated. Number in parenthesis represents percentage of sequence similarity.

| **Contig ID** | **Transcript Name** | **Length**  **(bp)** | **Protein Definition** | **Matched species and maximum identify (%)** | **E-value** | **Uniprot /**  **Gi number** |
| --- | --- | --- | --- | --- | --- | --- |
| Contig1 | TX01_VGDQ | 426 | Dinoponeratoxin Da-2501 | *Dinoponera australis* [ant]( 84,2) | 7.4x10^-02^ | 294863158 |
| Contig2 | TX02_VGDQ | 919 | CAP Venom allergen | *Solenopsis Invicta* [ant] (59,0) | 2x10^-80^ | 6136163 |
| Contig3 | HP03_VGDQ | 351 | Venom secreted hypothetical peptide | - | - | - |
| Contig4 | HP04_VGDQ | 767 | Hypothetical protein | *Harpegnathos saltator* [ant] (54,3) | 5x10^-58^ | 307212163 |
| Contig5 | TX05_VGDQ | 485 | ICK-like | *Androctonus amoreuxi* [scorpion] | 1.4x10^-03^ | PS01138 |
| Contig6 | TX06_VGDQ | 430 | Pilosulin | *Myrmecia banki* [ant] | 6x10^-3^ | Q68Y23 |
| Contig7 | HP07_VGDQ | 395 | Venom secreted hypothetical peptide | - | - | - |
| Contig8 | TX08_VGDQ | 184 | Altitoxin (?) | *Parabuthus transvaalicus* [scorpion] | 1.8 | PC1B5 |
| Contig9 | TX09_VGDQ | 578 | Dinoponeratoxin Da-3177 | *Dinoponera australis* [ant] (85,7) | 4x10^-07^ | 294863162 |
| Contig10 | - | 576 | No hit | - | - | - |
| Contig11 | CP11_VGDQ | 807 | Cytochrome c oxidase | *Camponotus vafer* [ant](69,0) | 2x10^-53^ | 359828649 |
| Contig12 | TX12_VGDQ | 585 | Chymotrypsin inhibitor | *Acromyrmex echinatior* [ant] | 1x10^-19^ | EGI67859 |
| Contig13 | HP13_VGDQ | 954 | Histone-lysine N-methyltransferase | *Hydra magnipapillata* [cnidarian] (76,9) | 5x10^-3^ | 449685068 |
| Contig14 | - | 357 | No hit | - | - | - |
| Contig15 | - | 388 | No hit | - | - | - |
| Contig16 | CP16_VGDQ | 804 | ATP synthase | *Vellenhovia benzai* [ant] (59,2) | 2x10^-19^ | 373432543 |
| Contig17 | TX17_VGDQ | 640 | PBP/GOBP Venom allergen 2/4-like | *Solenopsis saevissima* [ant] | 1x10^-4^ | A5X2H7 |
| Contig18 | CP18_VGDQ | 436 | 28S partial ribosomal RNA gene | *Dinoponera* sp. Pr01 voucher [ant] | 0.0 | DQ400983.1 |
| Contig19 | TX19_ VGDQ | 424 | Dinoponeratoxin Da-2501/Da-1585 | *Dinoponera australis* [ant] | 45 | P0CF01 |
| Contig20 | CP20_VGDQ | 717 | Mitochondrial DNA | mitochondrion *Pristomyrmex punctatus* [ant] | 2x10^-155^ | AB556947.1 |
| Contig21 | TX21_ VGDQ | 708 | PBP/GOBP Venom allergen 2/4-like | *Solenopsis invicta* [ant] | 7x10^-5^ | P35777 |

(?): Should be confirmed
