# Supplementary material for: Transcriptome Analysis in Venom Gland of the Predatory Giant Ant Dinoponera quadriceps: Insights into the Polypeptide Toxin Arsenal of Hymenopterans
Source: PLoS One. 2014 Jan 31;9(1):e87556. doi: 10.1371/journal.pone.0087556 (PMC3909188; doi:10.1371/journal.pone.0087556)
Supplement: Table S2 — Overview of experimental data from the next generation RNA sequencing. (DOC) [file pone.0087556.s002.doc]

### Table S2 – Overview of experimental data from the Next Generation RNA sequencing

| **Items** | **Number** |
| --- | --- |
| Total transcripts | 18,546 |
| Max contig length(bp) | 8114 |
| Min contig length(bp) | 101 |
| Whole dataset length(bp) | 4,304,312 |
| Average contig lengths(bp) | 232.09 |
| N50 | 249 |
| N90 | 119 |
