# Supplementary material for: Transcriptome Analysis in Venom Gland of the Predatory Giant Ant Dinoponera quadriceps: Insights into the Polypeptide Toxin Arsenal of Hymenopterans
Source: PLoS One. 2014 Jan 31;9(1):e87556. doi: 10.1371/journal.pone.0087556 (PMC3909188; doi:10.1371/journal.pone.0087556)
Supplement: Table S3 — Annotations of the identified venomous contigs in the D. quadriceps venom gland. Contigs representing candidate toxin-like peptides based on the alignment of EST data set, prediction of signal peptides and ORFs, and BLASTx comparison. GI denotes sequence identification number of “top hit” toxin homologous. Number in parenthesis represents percentage of sequence similarity. (DOC) [file pone.0087556.s003.doc]

## Table S3 - Annotation of identified venomous contigs in *D. quadriceps* venom gland

Contigs representing candidate toxin-like peptides based on the alignment of EST data set, prediction of signal peptides and ORFs, and BLASTx comparison. GI denotes sequence identification number of “top hit” toxin homologous. Number in parenthesis represents percentage of sequence similarity.

| **Contig ID** | **Length**  **(bp)** | **Protein Definition** | **Matched species and maximum identify (%)** | **E-value** | **Gi number** |
| --- | --- | --- | --- | --- | --- |
| *Contig2 | 483 | Dinoponeratoxin Da-3177 | Dinoponera australis [ant]( 85.71) | 8e-07 | 294863162 |
| *Contig1 | 464 | Dinoponeratoxin Da-2501 | Dinoponera australis [ant]( 84.21) | 3e-06 | 294863158 |
| *Contig12 | 1051 | Venom allergen III; Flags: Precursor | Solenopsis invicta [ant](56.58) | 2e-72 | 14424466 |
| *Contig27 | 1656 | Venom allergen I; Flags: Precursor | Solenopsis invicta [ant](35.51) | 1e-44 | 75006920 |
| Contig141 | 3365 | Protein lethal(2)essential for life | Acromyrmex echinatior [ant](93.33) | 2e-33 | 332022897 |
| Contig351 | 3845 | Protein lethal(2)essential for life | Harpegnathos saltator [ant](86.90) | 1e-37 | 307197364 |
| Contig455 | 1497 | Probable multidrug resistance-associated protein lethal(2)03659 | Harpegnathos saltator [ant](86.25) | 4e-169 | 307196484 |
| Contig857 | 966 | Probable multidrug resistance-associated protein lethal(2)03659 | Harpegnathos saltator [ant](76.36) | 3e-131 | 307196484 |
| Contig1700 | 826 | Probable multidrug resistance-associated protein lethal(2)03659 | Harpegnathos saltator [ant](81.82) | 3e-36 | 307196484 |
| Contig5586 | 139 | Lethal(2)neighbour of tid protein | Harpegnathos saltator [ant](86.96) | 5e-19 | 307207923 |
| Contig8914 | 165 | Lethal(2)neighbour of tid protein | Acromyrmex echinatior [ant](85.19) | 9e-19 | 332018132 |
| Contig11936 | 176 | Lethal(2)neighbour of tid protein | Harpegnathos saltator [ant](91.38) | 6e-23 | 307207923 |
| Contig12935 | 189 | Lethal(3)malignant brain tumor-like 3 protein | Harpegnathos saltator [ant](85.48) | 2e-09 | 307202837 |
| Contig17436 | 120 | Pre-mRNA-splicing regulator female-lethal(2)D | Harpegnathos saltator [ant](87.18) | 2e-11 | 307211429 |
| Contig17643 | 159 | Probable multidrug resistance-associated protein lethal(2)03659 | Harpegnathos saltator [ant](77.50) | 6e-10 | 307207958 |
| Contig18270 | 128 | Probable multidrug resistance-associated protein lethal(2)03659 | Harpegnathos saltator [ant](90.48) | 4e-14 | 307196485 |
| Contig187 | 1410 | PREDICTED: phospholipase A2 isozymes PA3A/PA3B/PA5 | Apis mellifera[honey bee](85.51) | 9.00E-109 | 328778177 |
| Contig662 | 1603 | Putative phospholipase B-like lamina ancestor | Harpegnathos saltator [ant](60.42) | 8e-26 | 307214880 |
| Contig4464 | 699 | Phospholipase A2 isozyme PA4 | Harpegnathos saltator [ant](88.89) | 2.00E-35 | 307198397 |
| Contig5423 | 186 | Phospholipase A2 isozyme PA3A/PA3B/PA5 | Acromyrmex echinatior [ant](83.93) | 2e-18 | 332029748 |
| Contig6851 | 234 | Lysophospholipase-like protein 1 | Harpegnathos saltator [ant](89.80) | 5e-17 | 307200032 |
| Contig9856 | 272 | Phospholipase A-2-activating protein | Harpegnathos saltator [ant](84.85) | 2e-22 | 307196404 |
| Contig10011 | 233 | Group XIIA secretory phospholipase A2 | Camponotus floridanus [ant](87.01) | 1e-34 | 307187781 |
| Contig12747 | 163 | Phospholipase B1, membrane-associated | Acromyrmex echinatior [ant](53.70) | 9e-06 | 332028550 |
| Contig13100 | 134 | Phospholipase B1, membrane-associated | Harpegnathos saltator [ant](75.61) | 3e-11 | 307193256 |
| Contig17494 | 145 | Phospholipase D3 | Camponotus floridanus [ant](97.92) | 6e-11 | 307176418 |
| Contig18196 | 185 | Phospholipase DDHD2 | Harpegnathos saltator [ant](91.80) | 8e-28 | 307211672 |
| Contig1144 | 495 | PREDICTED: U8-agatoxin-Ao1a-like isoform 1 | Bombus impatiens[bee](85.71) | 6e-39 | 350400650 |
| Contig326 | 451 | PREDICTED: venom carboxylesterase-6-like | Bombus terrestris[bee](56.14) | 2e-31 | 340722695 |
| Contig391 | 921 | PREDICTED: venom carboxylesterase-6-like | Apis florea[honeybee](63.25) | 3e-102 | 380027409 |
| Contig584 | 933 | PREDICTED: venom carboxylesterase-6 isoform 1 | Nasonia vitripennis [bee](69.77) | 1e-103 | 345497204 |
| Contig1381 | 150 | PREDICTED: venom carboxylesterase-6-like | Apis mellifera [honeybee](75.51) | 8e-13 | 48097744 |
| Contig1632 | 149 | PREDICTED: venom carboxylesterase-6-like | Megachile rotundata [bee](83.67) | 1e-16 | 383858940 |
| Contig3130 | 165 | PREDICTED: venom carboxylesterase-6-like | Megachile rotundata [bee](64.81) | 2e-12 | 383858940 |
| Contig3290 | 474 | PREDICTED: venom carboxylesterase-6 isoform 1 | Nasonia vitripennis [bee](64.84) | 2e-44 | 345497204 |

* represent contig was supported by the ESTs data.
