# Supplementary material for: Transcriptome Analysis in Venom Gland of the Predatory Giant Ant Dinoponera quadriceps: Insights into the Polypeptide Toxin Arsenal of Hymenopterans
Source: PLoS One. 2014 Jan 31;9(1):e87556. doi: 10.1371/journal.pone.0087556 (PMC3909188; doi:10.1371/journal.pone.0087556)
Supplement: Table S4 — Summary of toxin-like peptide candidates from the D. quadriceps venom gland transcriptome. Validation of some RNA-Seq assembled contigs according to the mapped EST fragments. Full-length open reading frames (ORFs) are indicated with a “+” sign. The presence or absence of a signal peptide in the ORFs is indicated by a “Y” (yes) or “N” (no). (DOC) [file pone.0087556.s004.doc]

## Table S4 - Summary of toxin-like peptide candidates from *D. quadriceps* venom gland transcriptome

Validation of some RNA-Seq assembled contigs according to the mapped EST fragments. Full-length open reading frames (ORFs) are indicated with a “+” sign. The presence or absence of a signal peptide in the ORFs is indicated by a “Y” (yes) or “N” (no).

| Contig ID | Length | Mapped EST fragments | ORF | Signal peptide |
| --- | --- | --- | --- | --- |
| Contig25 | 539 | 26 | + | Y |
| Contig3 | 2257 | 22 | + | N |
| Contig4 | 476 | 49 | + | Y |
| Contig10 | 254 | 20 | + | N |
| Contig5 | 482 | 34 | + | N |
| Contig8 | 675 | 16 | + | Y |
