# Supplementary figures and images for: Transcriptome Analysis in Venom Gland of the Predatory Giant Ant Dinoponera quadriceps: Insights into the Polypeptide Toxin Arsenal of Hymenopterans
Source: PLoS One. 2014 Jan 31;9(1):e87556. doi: 10.1371/journal.pone.0087556 (PMC3909188; doi:10.1371/journal.pone.0087556)

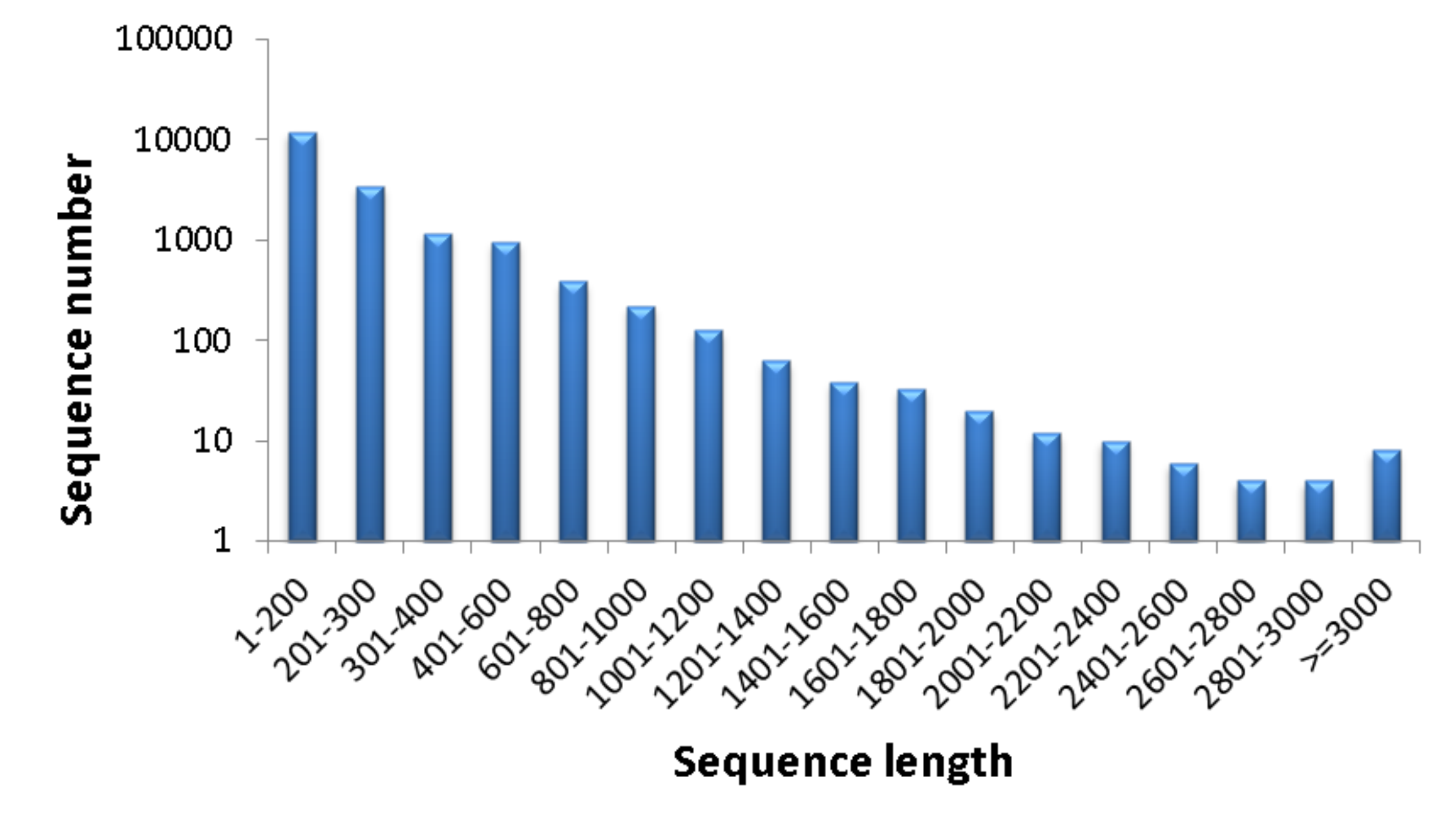

Supplement: Figure S1 — The length distribution of the assembled transcripts of D. quadriceps venom gland. Sequence length is expressed as base pairs (bp), and the frequency is expressed as the absolute number of sequences. The y-axis indicates the number of sequences of different lengths. (TIF) [file pone.0087556.s005.tif]

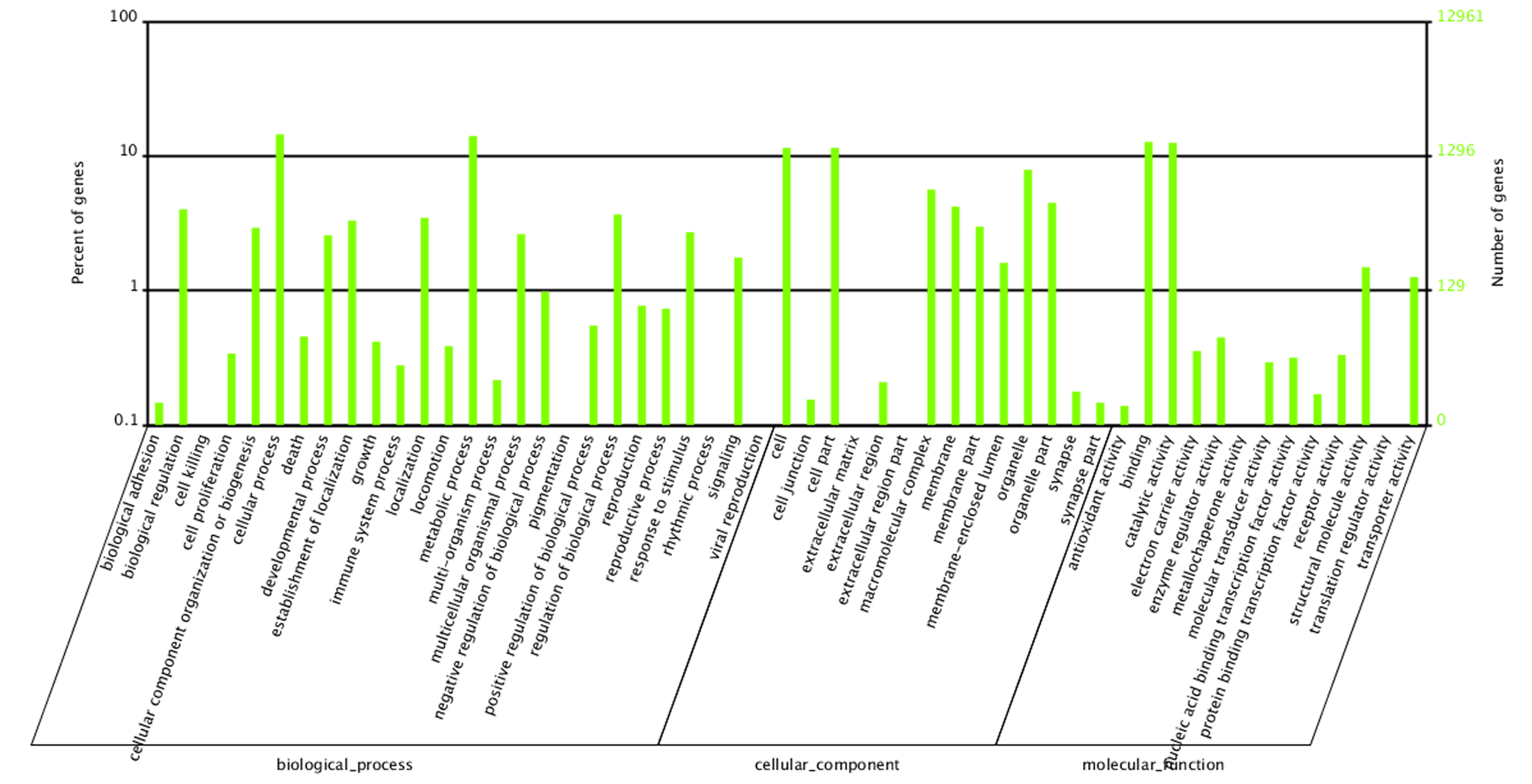

Supplement: Figure S2 — Gene ontology (GO) classification of contigs identified in the venom gland of D. quadriceps . Distribution of unigenes associated with biological processes, cellular components and molecular functions of the giant ant venome transcripts. (TIF) [file pone.0087556.s006.tif]

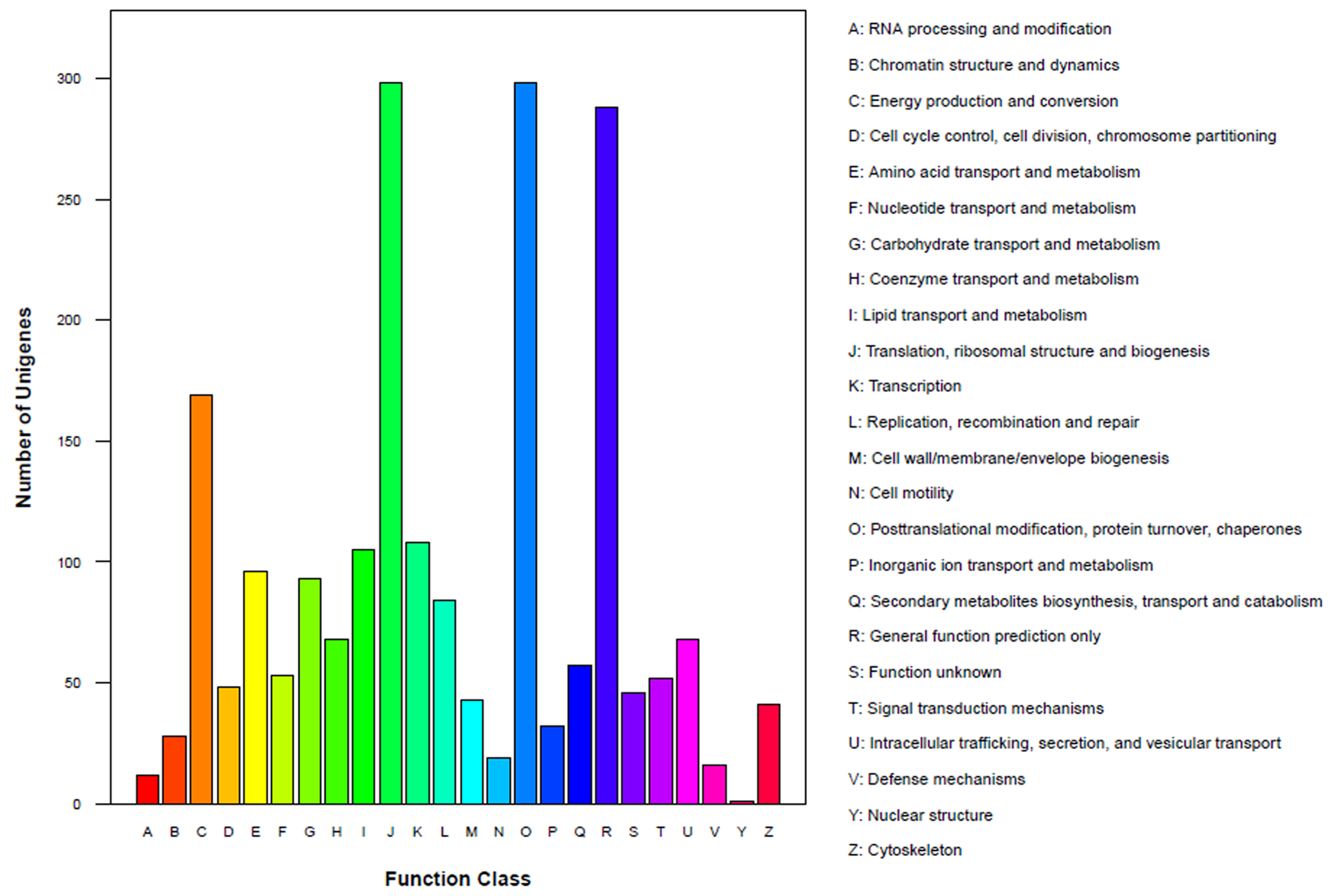

Supplement: Figure S3 — Clusters of orthologous groups (COG) function classification of the assembled giant ant venomic transcripts. Query of the COG database allowed for the classification of venom-related transcripts into 24 group based on functions (from A to Z) and attributes. The distribution of these groups of the D. quadriceps transcriptome (y-axis) is shown. (TIF) [file pone.0087556.s007.tif]

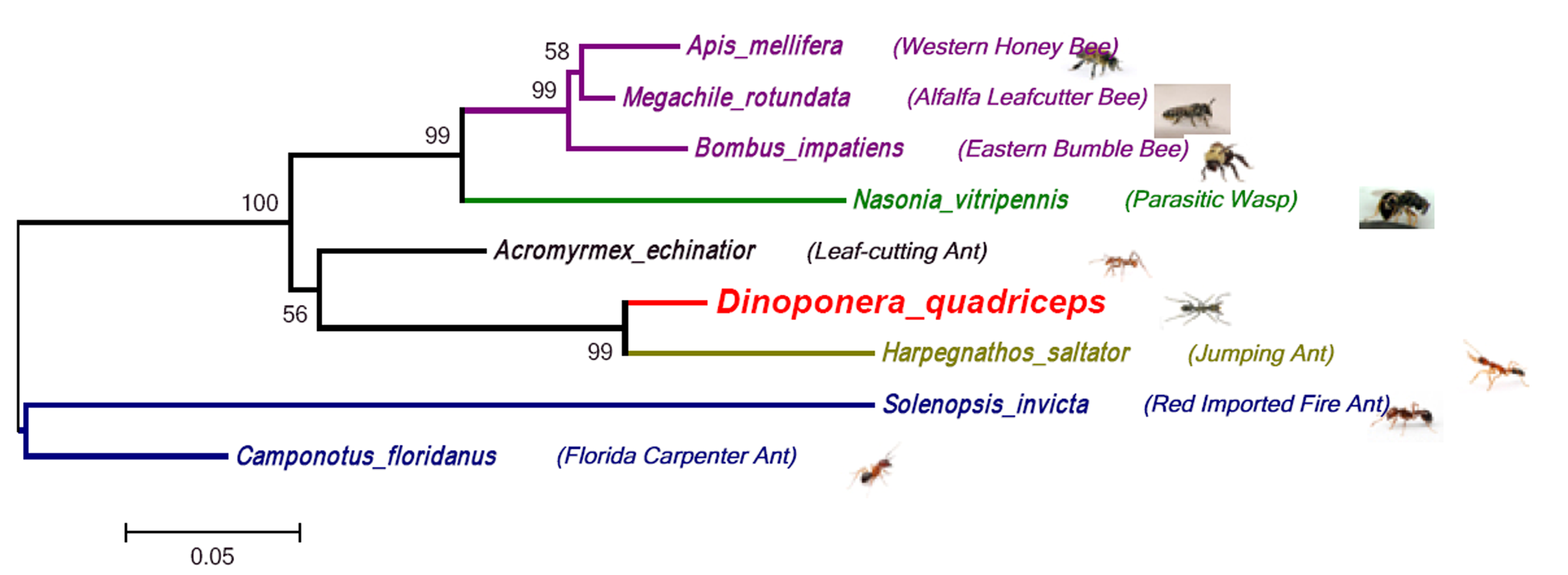

Supplement: Figure S4 — Comparison of sex determination proteins from D. quadriceps with their hymenopterans orthologs. Phylogenetic tree based on neighbor-joining analyses of a concatenated alignment of a sex determination protein and orthology relationships in multiple insects. The scale bar indicates 0.05 substitution per site. Apis mellifera, Megachile rotundata, Bombus impatiens, Nasonia vitripennis, Acromyrmex echinatior, Harpegnathos saltator, Solenopsis invicta, camponotus floridanus. (TIF) [file pone.0087556.s008.tif]

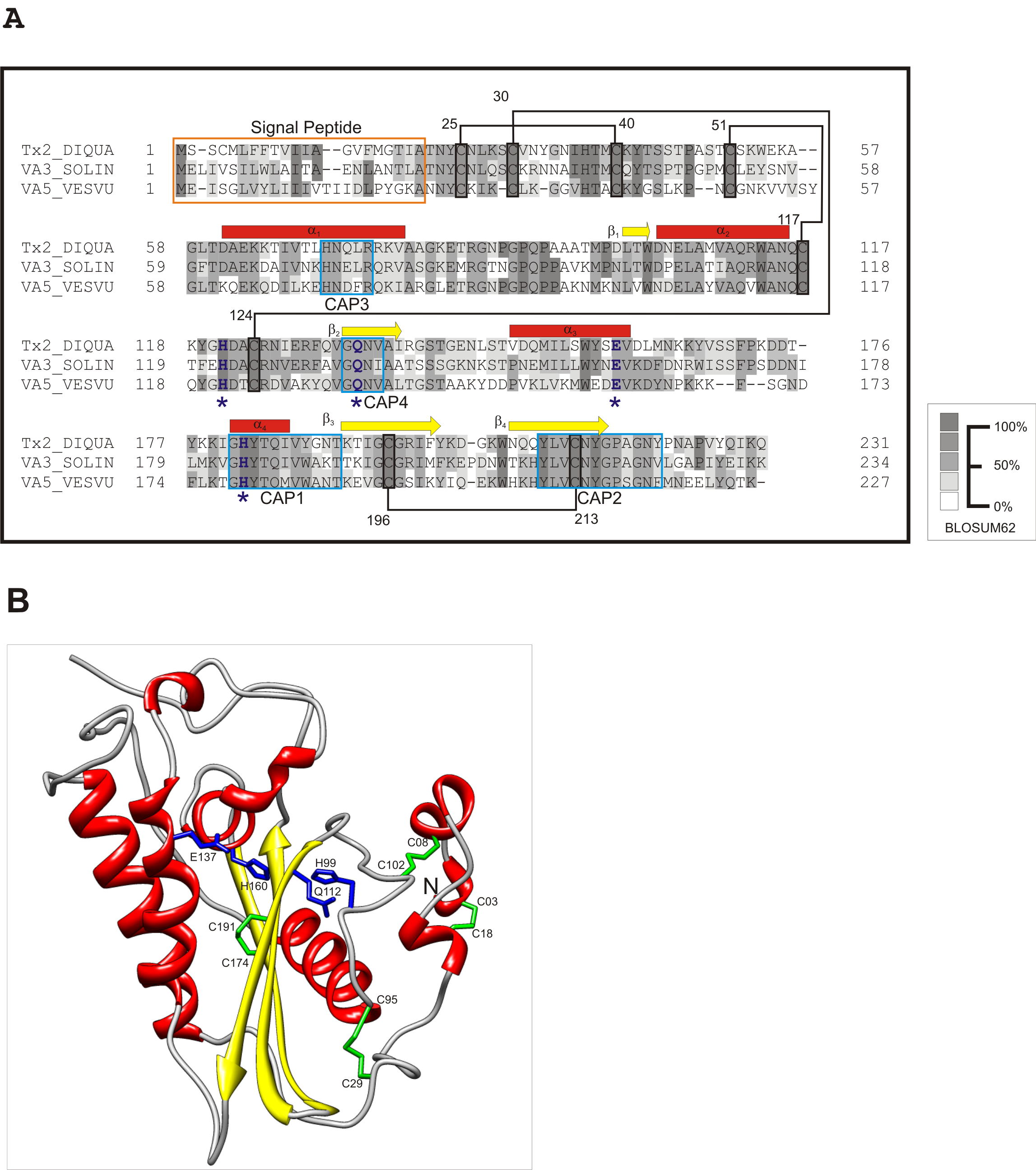

Supplement: Figure S5 — Structural comparison of D. quadriceps CAP venom allergen antigen 5 with two hymenopteran venom allergen and the tridimensional topology of CAP venom allergen antigen 5. The Dinoponera venom allergen precursor was aligned with the following Hymenoptera allergen Ag5 precursors using the BLOSUM62 scores: VA3_SOLIN (Solenopsis invicta venom allergen 3, Sol I 3) and VA5_VESVU (Vespula vulgaris Venom allergen 5, Ves5). Part A depicts the primary sequence analysis. The signal peptide is indicated by an orange box; the predicted pattern of disulfide bridges is represented by connected lines and numbered based on Dinoponera CAP Venom allergen 5 (dark gray box). The major conserved secondary structural elements are indicated by marks above the relevant amino acid sequences (red bar, α-helix; yellow arrow, β-strand). CAP signature motifs are marked by cyan boxes, and the histidine and glutamine/glutamic acid residues involved in the putative active site are also indicated (blue*). The histidines, which have the ability to form complexes with divalent cations, form a structure with some similarity to the protease active sites, which is consistent with the calcium-activated serine protease-like activity that has been reported for the Conus textile cysteine-rich venom protein endoproteinase Tex31 (Q7YT83). Part B: tri-dimensional structure based the on structural characteristics described in part A. (TIF) [file pone.0087556.s009.tif]

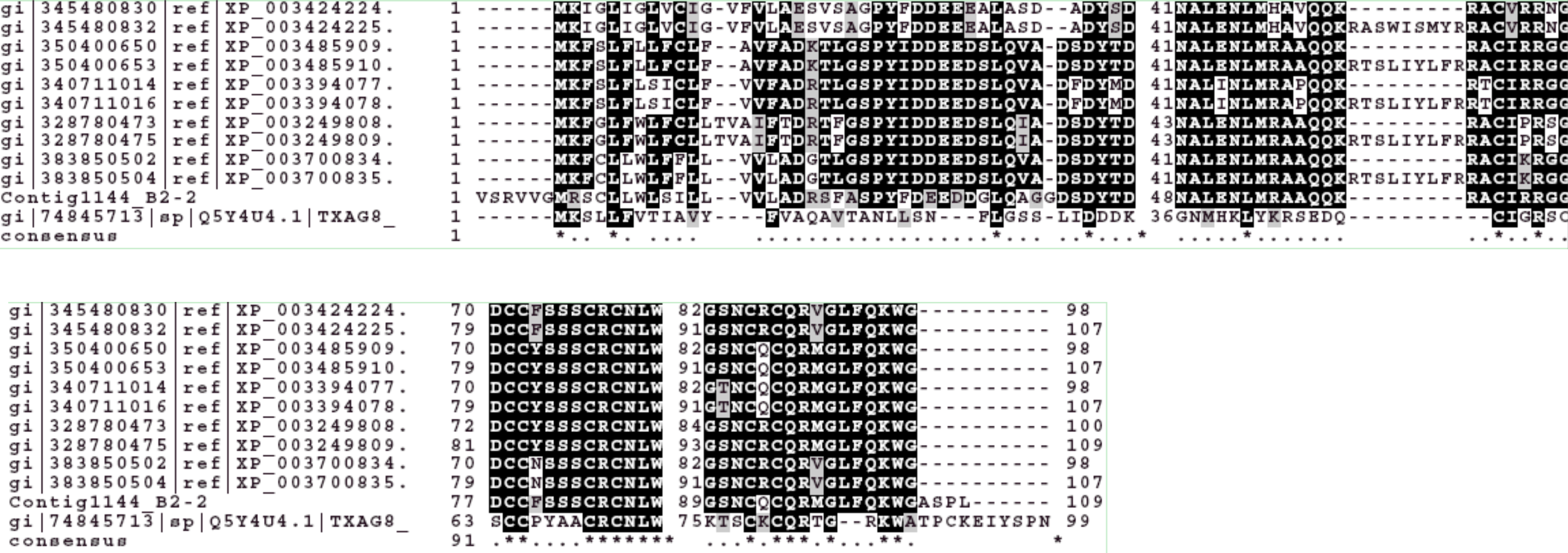

Supplement: Figure S6 — D. quadriceps U8-agatoxin-Ao1a-like venom homolog Contig1144_B2-2 was assembled with data from the RNA-Seq reads. TXAG8 (Q5Y4U4) is a U8-agatoxin-Ao1a-like venom homolog that was discovered by a program for the identification of toxin-related candidates in arthropod databases [81]. The other sequences came from query of the database for homologous entries. (TIF) [file pone.0087556.s010.tif]
